# Trafficking proteins show limited differences in mobility across different postsynaptic spines

**DOI:** 10.1016/j.isci.2023.105971

**Published:** 2023-01-13

**Authors:** Nikolaos Mougios, Felipe Opazo, Silvio O. Rizzoli, Sofiia Reshetniak

**Affiliations:** 1 Institute of Neuro- and Sensory Physiology, University Medical Center Göttingen, 37073 Göttingen, Germany; 2 Center for Biostructural Imaging of Neurodegeneration (BIN), University of Göttingen Medical Center, 37075 Göttingen, Germany; 3 NanoTag Biotechnologies GmbH, Göttingen, Germany

**Keywords:** Cell biology, Molecular biology, Neuroscience

## Abstract

The function of the postsynaptic compartment is based on the presence and activity of postsynaptic receptors, whose dynamics are controlled by numerous scaffolding, signaling and trafficking proteins. Although the receptors and the scaffolding proteins have received substantial attention, the trafficking proteins have not been investigated extensively. Their mobility rates are unknown, and it is unclear how the postsynaptic environment affects their dynamics. To address this, we analyzed several trafficking proteins (α-synuclein, amphiphysin, calmodulin, doc2a, dynamin, and endophilin), estimating their movement rates in the dendritic shaft, as well as in morphologically distinct “mushroom” and “stubby” postsynapse types. The diffusion parameters were surprisingly similar across dendritic compartments, and a few differences between proteins became evident only in the presence of a synapse neck. We conclude that the movement of trafficking proteins is not strongly affected by the postsynaptic compartment, in stark contrast to the presynapse, which regulates strongly the movement of such proteins.

## Highlights

• We use FRAP to analyze protein movement in mushroom and stubby synapses
• Trafficking proteins show similar mobility in different postsynaptic compartments
• The postsynaptic density does not affect the mobility of trafficking proteins
• The mushroom spine neck only has a mild effect on trafficking protein mobility

## Graphical abstract

**Figure:**
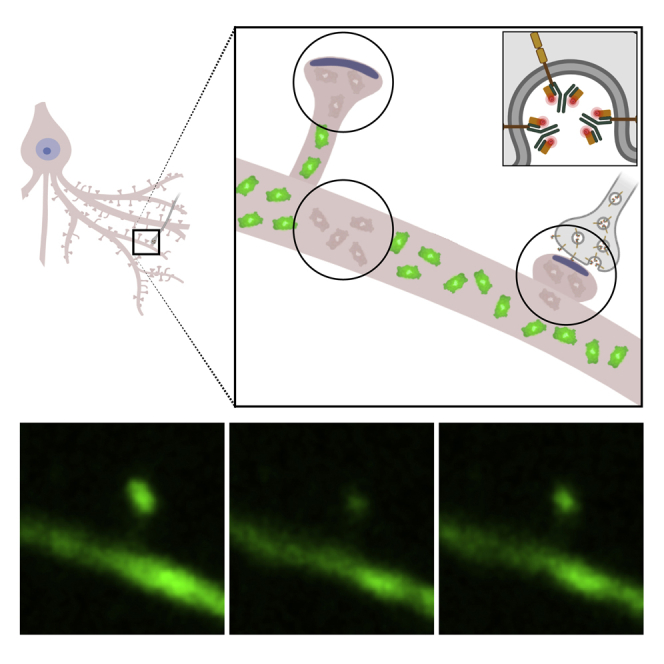

## Introduction

Complex subcellular compartmentalization is a fundamental feature of eukaryotic cells,^1^ and the correct organization of the protein makeup is crucial for the proper function of the respective compartments. Many subcellular compartments, including classical organelles such as the nucleus, Golgi complex or mitochondria, are surrounded by membranes and maintain their identity by being thus physically separated from the rest of the cell. Others, such as nucleoli, P bodies, centrosomes or stress granules, exhibit unique compositions in the absence of membrane separation.^2^ This situation is especially prominent in neurons, where synapses are known to have a unique composition, despitelacking membranes to separate them from the neuronal environment. The mechanisms for such segregation are still being investigated, with more insight being available on the presynaptic side. There, detailed organelle and protein compositions have been available for years,^3^^,^^4^^,^^5^ and the dynamics of many presynaptic proteins have been investigated.^6^^,^^7^^,^^8^ Taken together, these studies indicate that the behavior of the majority of proteins is largely affected by their interaction with the synaptic vesicle cluster,^9^ as many of presynaptic trafficking proteins are known to bind to the synaptic vesicles and other components of the cluster.^4^^,^^7^^,^^9^^,^^10^^,^^11^

Less information is available on possible segregation mechanisms at the postsynapse. The composition of the postsynapse has been less well understood than that of the presynapse, historically, but several studies focused recently on its protein organization,^12^ and the mobility of some postsynaptic proteins, including membrane receptors, scaffolds and cell adhesion components has been thoroughly investigated.^13^^,^^14^^,^^15^ Less is known about the mobility, organization, and regulation of soluble proteins, especially trafficking molecules. Some studies suggest that the PSD is organized via liquid-liquid phase separation,^16^^,^^17^^,^^18^ whereas other indicate existence of nanoclusters^19^^,^^20^^,^^21^ that might be formed and/or maintained by mechanisms independent of phase separation.^22^ Some of the postsynaptic trafficking molecules might interact strongly with receptors and PSD proteins in these nanodomains, whereas others might be regulated by different mechanisms. For example, dynamin has been reported to interact with the PSD protein Homer, to position components of the clathrin endocytosis machinery in the vicinity of the PSD.^23^ This was later confirmed by the observation of several trafficking proteins, including clathrin, dynamin and amphiphysin, at or near PSDs in cultured neurons.^24^ Overall, the most likely explanation is that a proportion of these molecules are able to organize in a stable structure bound to the PSD, the endocytic zone,^24^ which leaves the dynamics of the non-bound, soluble proteins unclear. They could be governed by PSD interactions, or could simply be driven by the dendritic spine neck acting as a diffusion barrier.^25^^,^^26^^,^^27^^,^^28^^,^^29^ At the same time, it remains unclear how strongly the neck affects the dynamics of different molecules, and how compartmentalization is achieved in the neck-less spines. Because trafficking molecules in both the pre- and the postsynapse can interact with the major determiners of these compartments (the synaptic vesicle cluster and the PSD, respectively), a question arises whether the regulation of the mobility of the trafficking molecules would be similar in both cases, or if it is compartment-specific. The key to understanding the organization of the trafficking proteins in relation to the PSD would be to study neck-less spines, thus separating the effects of the PSD from the gate effect of the spine neck.

To test this, we analyzed here the mobility of 6 soluble proteins in dendritic shafts and in spines with or without a neck structure (mushroom and stubby spines, respectively). We analyzed primary hippocampal neurons using fluorescence recovery after photobleaching (FRAP), and we found very limited differences in protein mobility between shafts and spines, indicating that the presence of the PSD does not affect drastically the behavior of the trafficking molecules. Some differences between proteins could be observed in mushroom spines, hinting that the morphological gate (the spine neck) plays a more important role in determining protein mobility than the presence of the PSD. This is in strong contrast to the presynapse, where no gate effects could be observed, whereas the SV cluster controlled the dynamics of most proteins, as mentioned above.^7^

## Results

### A FRAP-based procedure for trafficking molecule analysis

**Figure 1:**
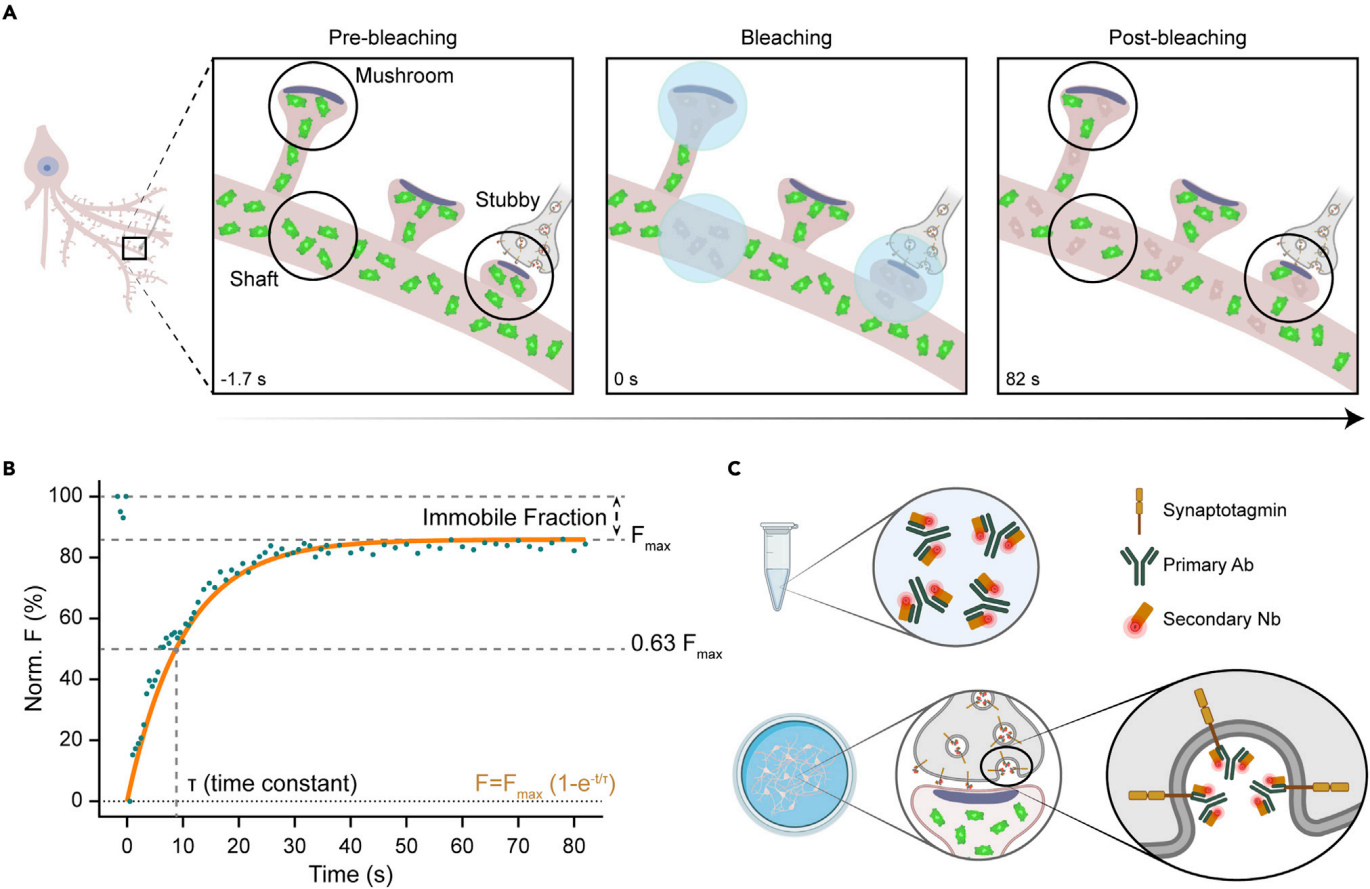
Schematic representation of the FRAP experiments (A) Three different neuronal compartments (mushroom spines, stubby spines and dendritic shafts) were analyzed. mEGFP-tagged molecules (green) in a defined region were photobleached and the fluorescent recovery of the same region was monitored by confocal live imaging. (B) The fluorescence intensity recorded during the movie is fitted with a one-phase exponential association curve. The time constant of recovery and the immobile fraction (fraction of bleached molecules that were not replaced) are calculated from the fit. (C) Single-step live immunolabeling of synaptotagmin 1, for the identification of stubby spines. Primary antibodies (Ab) and secondary fluorescent nanobodies (Nb-AbberiorSTAR 635p) were mixed in a tube before their incubation with neurons.^30^ Active synapses without a neck structure are observed by the colocalization of mEGFP and synaptotagmin signals.

**Figure 2:**
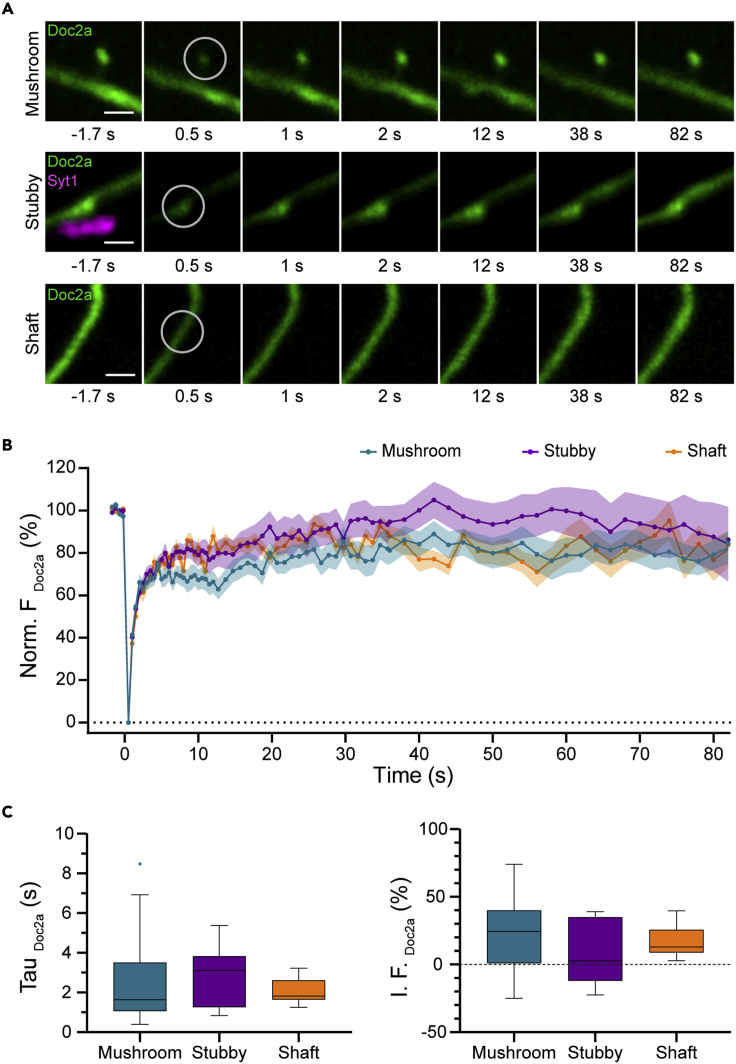
Representative results and analysis process (A) Exemplary images from different compartments (mushroom spine, stubby spine and dendritic shaft) of a neuron expressing doc2a tagged with mEGFP, during FRAP experiments. Circled regions were used for analysis. Stubby spines were localized on synapses after labeling against synaptotagmin 1. The experiments were performed at DIV11. Scale bar: 1 μm. (B) Recovery curves from the three neuronal compartments indicated in A. The symbols show mean ± SEM; N (mushroom) = 25, N (stubby)= 10, N (shaft) = 11. (C) Boxplots of time constants (Tau) and immobile fractions (I.F.) of doc2a in mushroom spines, stubby spines and dendritic shafts. In boxplots, the middle line shows the median, the box edges indicate the 25th and 75th percentile and the error bars show the min and the max values. N (mushroom) = 25, N (stubby)= 10, N (shaft) = 11.

To analyze protein mobility, we relied on FRAP experiments in primary hippocampal neurons overexpressing different proteins of interest fused to mEGFP (monomeric enhanced green fluorescent protein). We measured FRAP behavior in dendritic shafts and spines of two types: mushroom and stubby (Figures 1A and 1B). Although mushroom spines can be easily identified by their morphology, we needed an additional step to localize synapses formed by stubby spines, by labeling their respective presynaptic terminals. For this, we used primary antibodies against the luminal domain of an SV protein, synaptotagmin (Syt 1), in combination with secondary nanobodies, following a single-step live immunolabeling procedure (Figure 1C). After locating the synaptotagmin signal, we performed FRAP experiments on the stubby spines opposed to it (Figure 2A, middle).

Because protein overexpression can affect protein dynamics (for example, by saturation of binding sites), we took precautions to minimize such artifacts. The transfection procedure we used has been described previously,^7^ and ensures mild overexpression levels, which do not affect the protein mobility behavior in the presynapse. In agreement with our previous work, the overexpression levels in the current study are low, typically below 2-fold increases in protein abundance, and do not affect the synapse size (Figures S1–S6). We also found no significant correlation between the molecular weights of the proteins tested and their mobility parameters (P_Taus_> 0.27 and P_I.F._> 0.07, Figure S7), indicating that protein mobility is mostly controlled by specific interactions rather than by size, which would have been the case if overexpression was strong enough to lead to saturation of binding sites. We therefore do not expect significant effects of overexpression on the protein mobility measured in this work.

### FRAP investigations in different spine types

After validating the imaging procedure, we analyzed the mobility of various proteins in the postsynaptic compartments. The mobility of many transmembrane proteins in the postsynapse has already been studied (Choquet and Triller, 2013; Obashi et al., 2021; Rusakov et al., 2011). Here we focused on soluble proteins, choosing molecules involved in signaling and membrane trafficking, with the aim to also compare their behavior in the post- and presynapse. The list of analyzed proteins includes amphiphysin, endophilin, dynamin, α-synuclein, calmodulin, and doc2a. Amphiphysin and endophilin contain BAR and SH3 domains that promote membrane curvature formation during the early stages of endocytosis and also participate in recruiting clathrin and dynamin to the endocytic sites.^31^^,^^32^ Dynamin is a GTPase that acts on the neck of the clathrin-coated vesicle and induces membrane scission.^31^ Calmodulin and doc2a are calcium sensors involved in the regulation of multiple cellular processes, including exocytosis and receptor trafficking.^33^^,^^34^ Finally, α-synuclein is an intrinsically disordered, lipid-binding protein, primarily involved in the regulation of exocytosis events and possibly also other steps in the secretory pathway.^35^

An example of FRAP results obtained from the calcium sensor protein doc2a, is shown in Figure 2. The mobility behavior of doc2a in different postsynaptic compartments did not differ substantially (Figures 2B and 2C). The same general trend is observed for almost all analyzed proteins (Figures S8–S14), suggesting that the presence of the synaptic components does not impose a strong effect on the mobility behavior of the tested proteins. The only notable exceptions are the BAR-domain proteins amphiphysin and endophilin. Amphiphysin has a significantly slower recovery and smaller immobile fraction in stubby spines than in mushroom spines or dendritic shafts (Figure S9), whereas endophilin recovers more slowly in mushroom spines compared to stubby spines or dendritic shafts (Figure S13). These differences in mobility are probably because of the capacity of these proteins to interact with curved sites on the plasma membrane, which are abundant in synapses. BAR domain proteins are known to bind sites with different curvatures, and in spines they participate in synapse initiation and remodeling,^36^ which offers a potential explanation for the different behaviors of amphiphysin and endophilin.

**Figure 3:**
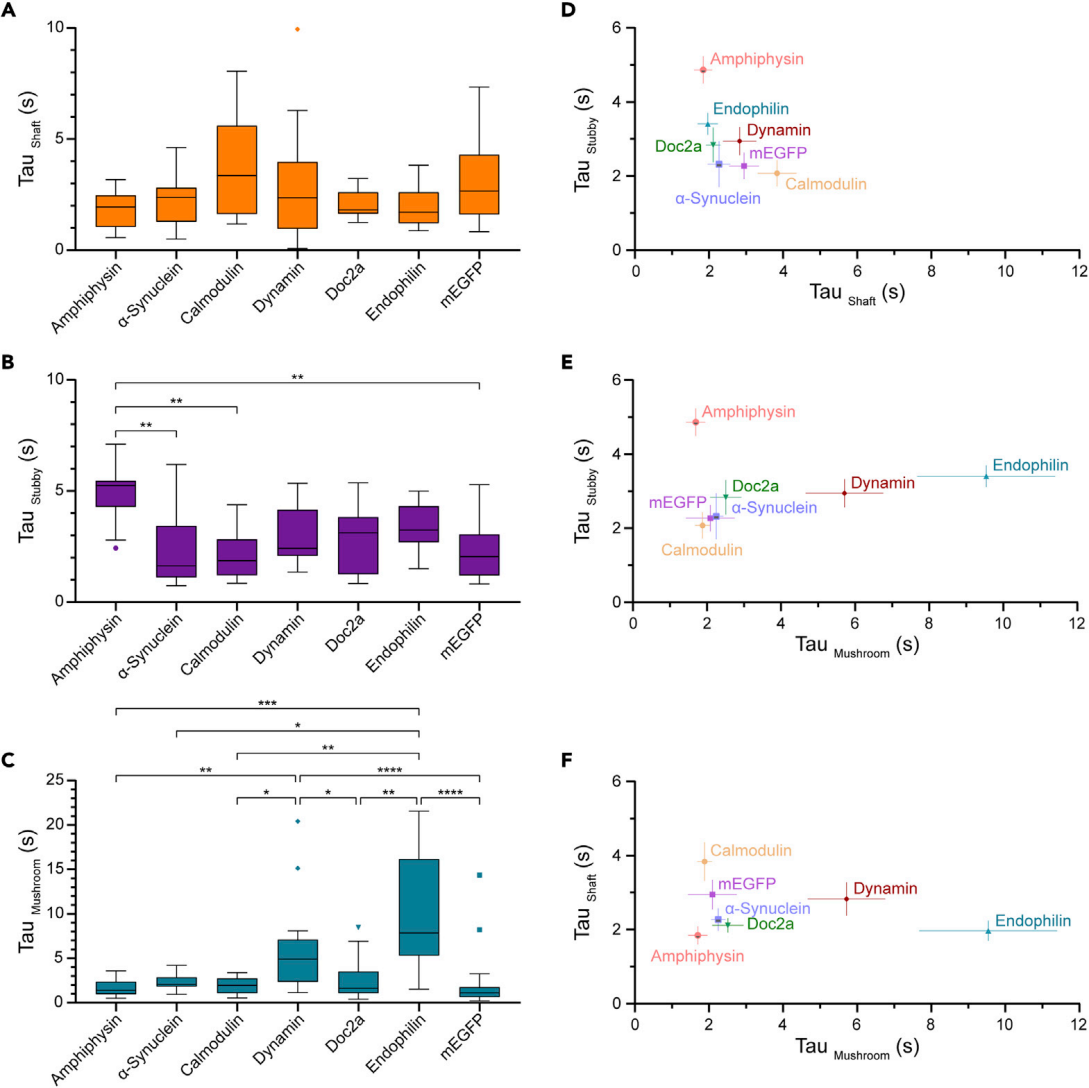
Recovery rate comparisons of different proteins in three postsynaptic compartments (A–C) Boxplots of time constants (Tau) in mushroom spines (A), stubby spines (B) and dendritic shafts (C) of α-synuclein, amphiphysin, calmodulin, doc2a, dynamin, endophilin and mEGFP. Asterisks indicate statistical significance after Kruskal-Wallis tests, followed by multiple comparisons with Dunn’s correction, ∗p<0.05, ∗∗p<0.01, ∗∗∗p<0.001, ∗∗∗∗p<0.0001. (D–F) Time constants (Tau) of all analyzed proteins in mushroom spines and dendritic shafts (D), in mushroom spines and stubby spines (E), in stubby spines and shafts (F). Symbols show mean ± SEM. In boxplots, the middle line shows the median, the box edges indicate the 25th and 75th percentile and the error bars show the min and the max values.

When comparing the behaviors of different proteins, we also found very limited differences in mobility in the shafts (Figures 3A and S15A) and stubby spines (Figures 3B and S15B). The only differences were observed for amphiphysin, which was less mobile in the stubby spines than our smallest and, in principle, most mobile proteins, α-synuclein and calmodulin. More profound differences between proteins could be observed in the mushroom spines (Figures 3C and S15C). Endophilin and dynamin are significantly less mobile in this compartment, compared to all other tested proteins, possibly because of their dimerization, which enlarges the overall size of the moving particles, thereby enhancing the gate effect of the spine neck (albeit this effect is not observed for amphiphysin, which is also expected to form dimers.^37^^,^^38^

### The pre- and postsynapse exhibit contrasting mechanisms

**Figure 4:**
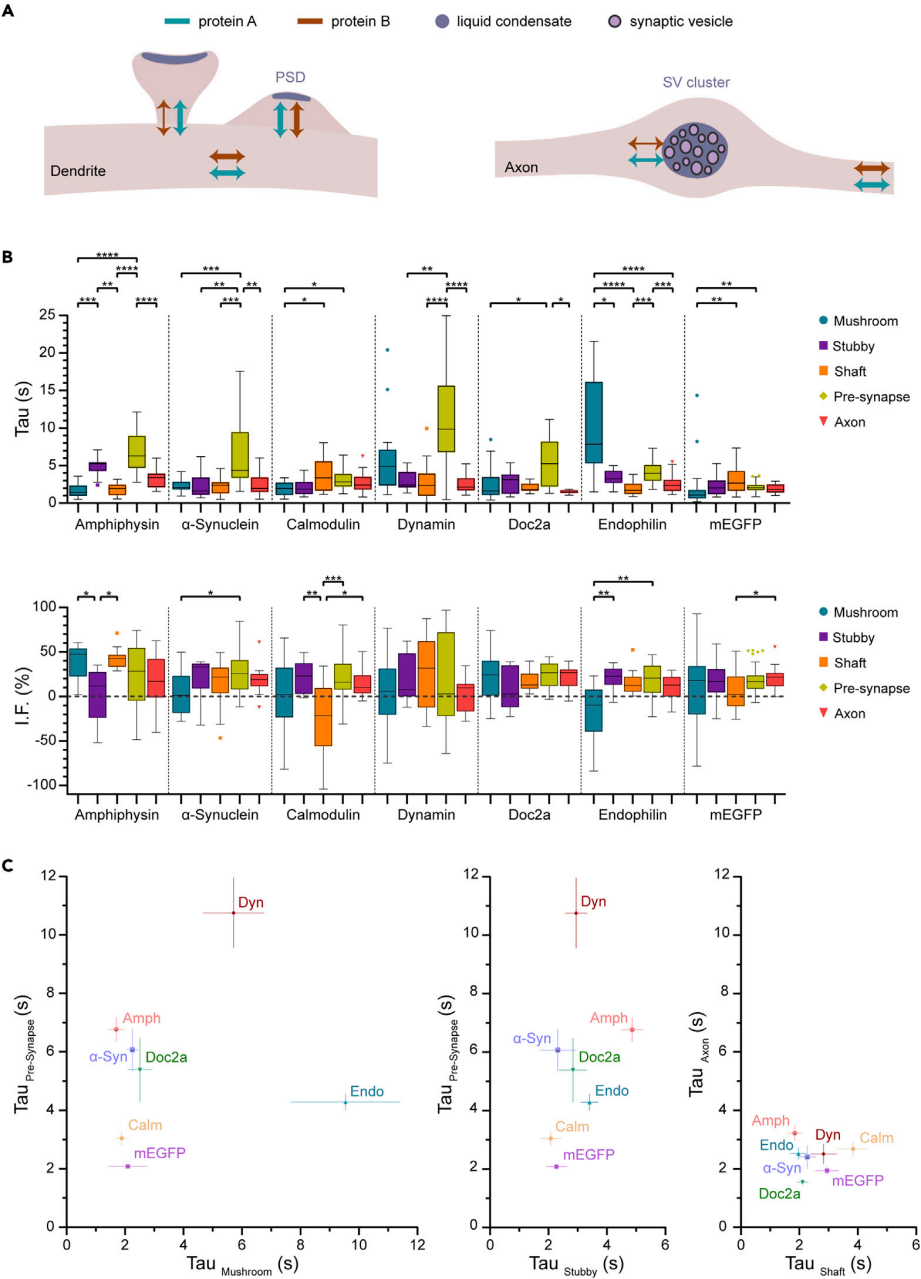
A comparison between pre- and postsynaptic protein mobility (A) Scenarios for protein mobility of two proteins in the post- and presynaptic compartments. LLPS-controlled regions, such as the postsynaptic density (PSD) and SV cluster can induce changes in protein kinetics. In the postsynapse (left) no such effect is observed for stubby spines, where protein exchange does not differ significantly between proteins and is similar to that in the dendritic shaft (illustrated by arrows of the same thickness). Only in mushroom spines differences between proteins become apparent. In presynapses (right), on the contrary, all proteins are slowed down in the SV cluster, with substantial differences observed between different proteins and between synapses and axons. (B) Time constants and immobile fractions (I.F.) of all analyzed proteins in post- and presynaptic compartments (mushroom spines, stubby spines, dendritic shafts, presynapse and axon). The middle line shows the median, the box edges indicate the 25th and 75th percentile and the error bars show the min and the max values. Asterisks indicate statistical significance after Kruskal-Wallis tests, followed by multiple comparisons with Dunn’s correction, ∗p<0.05, ∗∗p<0.01, ∗∗∗p<0.001, ∗∗∗∗p<0.0001. Data of analyzed proteins in axons and presynapses were retrieved from.^7^ (C) Scatterplots of time constants (Tau) from all analyzed proteins in presynapses and mushroom spines, in presynapses and stubby spines, and in axons and dendritic shafts. Symbols indicate mean ± SEM.

The data discussed in the previous paragraphs indicates that the presence of the PSD in the stubby spines has little effect on the mobility of most trafficking molecules, in strong contrast to what happens in the presynapse, where the SV cluster strongly influences protein mobility (Figure 4A). Previously, we have analyzed the mobility of a wide selection of proteins in the presynaptic compartment by relying on the same exact imaging and analysis procedure as used here.^7^ This provided us with the opportunity to compare directly the kinetic parameters obtained here with those from our previous work. We already compared protein recovery parameters in presynaptic boutons to the recovery in axonal regions of comparable size, and found that almost all proteins exhibit slower recovery in the synaptic boutons compared to axons.^7^ In contrast, such an effect was not observed here when the postsynapses were compared to the neighboring dendritic regions (shaft) for the same proteins (Figures 4B and 4C).

α-Synuclein, dynamin, and doc2a show few significant differences in their behaviors in various postsynaptic compartments, and tend to move faster there than in the presynapse, whereas all three proteins move significantly slower in synaptic boutons than in axons. Calmodulin shows a similar behavior, albeit its synaptic bouton mobility is only slightly slower than its axonal mobility. Of interest, amphiphysin moves more slowly in the stubby spines than in the mushroom spines or the shaft. Another noteworthy protein is endophilin, which behaves in the opposite fashion: it moves more slowly in the mushroom spines, while also having a larger mobile fraction there. It is difficult to pinpoint the direct causes of these differences, but they may be linked to specific interactions of these BAR-domain proteins and the synaptic curvature, as already noted above.

In general, most proteins analyzed here also move substantially faster in the postsynapses than in the presynapses (Figures 4B and 4C). We found no correlation in their mobility in pre- and postsynapses (Figure 4C), suggesting that different regulatory mechanisms are in place in these two compartments (mentioned in more detail in the discussion).

Overall, this overview of pre- and postsynaptic mobility serves to underline once more the limited differences in mobility observed between proteins in different postsynaptic compartments, whereas the presynaptic compartment, through the SV cluster, exerts a strong influence on protein mobility.

## Discussion

Our results show surprisingly limited differences between protein mobility in different postsynaptic compartments, indicating that the postsynaptic environment, including the presence of the PSD, has little effect on the mobility of trafficking molecules. Some differences between proteins were observed in mushroom spines, but not in stubby spines, suggesting that the spine neck provides the strongest effect of the postsynaptic compartment on protein mobility.

Compartmentalization of dendritic spines has been discussed for decades, and the synaptic neck has been suggested to be the main barrier regulating the postsynaptic environment, including the distribution of calcium ions,^39^^,^^40^ consequently affecting synaptic plasticity.^27^ The effects of other spine components, as the PSD, could not be investigated in isolation, because studies aiming to analyze spine compartmentalization focused on mushroom spines, and rarely analyzed stubby spines. In the absence of a dendritic neck, one other prominent structure of the spine could control the behavior of the trafficking proteins, namely the PSD. As already mentioned in the introduction, some endocytic proteins have been shown to bind directly to PSD components, resulting in the accumulation of multiple such molecules at perisynaptic sites, in the so-called endocytic zone, in the immediate vicinity of the PSD.^23^^,^^24^

Although often seen as immature, stubby spines contain the same proteins, and in the same average amounts, as the mushroom spines.^12^ Our work suggests that trafficking molecules are not significantly affected by the postsynaptic environment in stubby spines, and that, although we found some differences between proteins in mushrooms spines, the presence of the neck also has only a limited effect. Previously the dendritic neck has been shown to restrict diffusion of membrane receptors,^41^^,^^42^^,^^43^ an effect that might be caused by both the neck geometry and its specific protein composition, as its cytoskeletal elements.^44^^,^^45^ The neck morphology is thought to contribute to the electrical and diffusional compartmentalization of the dendrites, which are also affected by synaptic plasticity, hinting that the spine compartmentalization does not solely depend on the synaptic neck geometry.^29^^,^^46^^,^^47^ In addition to restricting the diffusion of soluble molecules,^48^ the spine neck was found to contribute to the compartmentalization of signaling activities.^49^^,^^50^^,^^51^^,^^52^ In our work we find that the synaptic neck primarily affects BAR domain-containing proteins (Figure 4B). This is likely to be caused by the geometry of the plasma membrane, which would affect the degree of association of these proteins with the membrane, and not by the shape or size of the spine neck itself. Endophilin, for example, is known to be required for the formation of the spine head,^53^ therefore its slower recovery in the mushroom spines is expected and is explained by its association with areas of specific curvature of the spine head.

Overall, we can state that protein mobility was not substantially hindered in any type of postsynaptic spine, unlike in the presynaptic vesicle cluster, where cytosolic protein diffusion rates are, on average, 8-fold lower than those in the axon.^7^ The answer as to why such a drastic difference between the pre- and postsynapse exists probably lies in their differences in the usage of these proteins. Although postsynapses employ frequently the endocytic recycling of receptors, they are less reliant on trafficking molecules than the presynapse. In this compartment, the SVs undergo constant recycling, with dozens of release events happening each minute. It has been estimated that up to 10% of all clathrin available in the presynapse is required to retrieve one vesicle.^5^^,^^31^ It is therefore not surprising that mechanisms to restrict the diffusion of clathrin and other cofactors out of the presynapse must exist, to avoid the loss of such molecules, which would trigger inefficient endocytosis. To avoid this, multiple interactions between soluble proteins and SVs converge, leading to the formation of the stable SV cluster,^54^ which regulates the presence and function of the trafficking molecules.

In the postsynapse, on the contrary, the recycling of membrane proteins is much less frequent. The available trafficking proteins have been estimated to be able to sustain nearly 100-fold more endocytosis events than ever reported for any mushroom or stubby postsynapse.^12^ This compartment therefore does not have a stringent need to maintain a high availability of trafficking proteins in the vicinity of the PSD. In the absence of highly time-sensitive exo- and endocytosis events, there is also no need to restrict the diffusion of trafficking cofactors in or out of the synapse, which would explain the lack of differences in the FRAP recovery behavior between shafts and spines. Similarly, rates of protein mobility would have different effects on synaptic activity in pre- and postsynapses. Repeated stimulation has been estimated to quickly deplete the available pool of clathrin in the presynapses,^55^ leading to inefficient endocytosis. An increase of clathrin mobility rates could compensate for such a clathrin deficit, allowing higher activity rates. In the postsynapse no such bottlenecks exist for the endocytic machinery,^12^ therefore mild changes in trafficking protein mobility would have little to no effect on neurotransmission.

Historically, the pre- and postsynapse have often been correlated, and are thought to share several functional principles. Both compartments are involved in the process of neurotransmission and are dependent on endocytic recycling of SVs or receptors. Despite these similarities, our results show that they have very different behaviors in relation to the trafficking machinery. These differences are likely to extend beyond just protein mobility, highlighting that further research is needed to understand the differences between these related compartments, especially in regard to the functional regulation of the trafficking proteins in the postsynaptic compartment.

Onetype of the dendritic spines we did not analyze is thin spines. These spines are similar to stubby spines in their high dynamics and short lifetimes,^56^^,^^57^^,^^58^ but, as their name implies, have a thin neck-like structure, which presumably would contribute to their diffusional compartmentalization. It is difficult to predict how the trafficking molecules would behave in such spines. Most likely BAR-domain containing proteins would have dynamics different from both stubby and mushroom spines, because of the different membrane curvatures that they would interact with. Considering how highly dynamic thin spines are and their lack of a PSD, it is also likely that diffusion of other soluble molecules would not be significantly confined to these spines. However, it would be difficult to test these hypotheses in our experiments because of the short lifetimes of such spines.

Previous data showed a correlation between spine shapes and the trafficking proteins amounts, when comparing mushroom spines and stubby spines,^12^ suggesting that mushroom spines have better-organized trafficking machineries, presumably to keep up with the changes needed for plasticity. The stubby spines had trafficking protein amounts that were poorly organized, often not correlated with the synaptic weight. In our work, we found similar mobility rates in both compartments, which is probably a compensatory mechanism that allows the stubby spines to maintain their dynamics, despite having less well-organized endocytic machineries.

To conclude, our results show that the postsynaptic environment has a limited effect on the mobility of most soluble trafficking molecules in mushroom and stubby spines. Of all analyzed proteins, only three (amphiphysin, endophilin and dynamin) had mobility parameters slightly different from any other proteins, and only amphiphysin and endophilin had different behaviors between mushroom and stubby spines. Although contradicting the previous notion of the strong diffusional compartmentalization caused by the synaptic neck in mushroom spines, these results are in line with the absence of functional bottlenecks in the availability of trafficking proteins, which removes the need to confine protein diffusion to the spines.

### Limitations of the study

The experiments, as indicated above, were performed using protein overexpression. This may cause an over-estimation of protein mobility rates, because not all of the proteins of interest will be able to find their functional partners owing to a saturation of binding sites. Our analysis shows that the observed overexpression levels were mild, and therefore are not expected to drastically affect protein mobility. Nonetheless, the provided recovery rates should be considered as maximal mobility estimates. At the same time, the usage of FRAP introduces another caveat: in these experiments we only analyze populations, not single molecules, thus missing the potential heterogeneity of the molecule motion. Another limitation of the current study is the number of analyzed proteins, which is not as extensive as in some of our previous studies.^5^^,^^7^^,^^12^ In addition, the spines are not imaged in super-resolution, so their morphology is not described in full detail. Nevertheless, all of these caveats leave the main conclusions unaffected.

## STAR★Methods

### Key resources table

**Table:** 

REAGENT or RESOURCE	SOURCE	IDENTIFIER
**Antibodies**		

FluoTag®-X2 anti-Rabbit IgG	NanoTag Biotechnologies, Göttingen, Germany	Cat#N2402-Ab635P-S
FluoTag®-X2 anti-Mouse Ig kappa light chain	NanoTag Biotechnologies, Göttingen, Germany	Cat#N1202-Ab635P-S
FluoTag®-X2 anti-PSD95	NanoTag Biotechnologies, Göttingen, Germany	Cat#N3702-Ab580-L
Anti-alpha/beta Synuclein rabbit polyclonal antibody	Synaptic Systems, Göttingen, Germany	Cat#128 002; RRID:AB_887857
Anti-Amphiphysin rabbit polyclonal antibody	Synaptic Systems, Göttingen, Germany	Cat#120 002; RRID:AB_887690
AntiCalmodulin rabbit monoclonal antibody	Novus Bioscience, Wiesbaden-Nordenstadt, Germany	Cat#NB110-55649; RRID:AB_2243917
Anti-Doc 2a/b rabbit polyclonal antibody	Synaptic Systems, Göttingen, Germany	Cat#174 203; RRID:AB_11064600
Anti-Dynamin mouse monoclonal antibody	BD Bioscience, Heidelberg, Germany	Cat#610245; RRID:AB_397640
Anti-Endophilin 1 rabbit polyclonal antibody	Synaptic Systems, Göttingen, Germany	Cat#159 002; RRID:AB_887757
Anti-Synaptotagmin 1 mouse monoclonal antibody - luminal domain	Synaptic Systems, Göttingen, Germany	Cat#105 311; RRID:AB_993036

**Critical commercial assays**		

Lipofectamine™ 2000 Transfection Reagent	Sigma-Aldrich	Cat#11668030

**Experimental models: Cell lines**		

Rat primary hippocampal neurons	University Medical Center Göttingen	N/A

**Recombinant DNA**		

pEGFP-N1–α-Synuclein	University Medical Center Göttingen,^7^	N/A
pcDNA3.1-Amphiphysin-mEGFP	University Medical Center Göttingen,^7^	N/A
pcDNA3.1-Calmodulin 1-mEGFP	University Medical Center Göttingen,^7^	N/A
pEGFP-N1-Doc2a	University Medical Center Göttingen,^7^	N/A
pcDNA3.1-Dynamin 1-mEGFP	University Medical Center Göttingen,^7^	N/A
pcDNA3.1-Endophilin A1-mEGFP	University Medical Center Göttingen,^7^	N/A

**Software and algorithms**		

MATLAB and Statistics Toolbox Release R2016b	The MathWorks, Inc., Natick, Massachusetts, United States	N/A
GraphPad Prism version 9.1.2	GraphPad Software, San Diego, California USA	N/A
Custom scripts	This paper, deposited at https://figshare.com/	Figshare.com: https://doi.org/10.6084/m9.figshare.21732167

### Resource availability

#### Lead contact

Further information and requests for resources and reagents should be directed to and will be fulfilled by the lead contact Silvio O. Rizzoli (srizzol@gwdg.de).

#### Materials availability

This study did not generate new unique reagents.

### Experimental model and subject details

Primary hippocampal neurons were obtained from postnatal day 0–3 rats (Wistar, wild-type, *Rattus norvegicus*) and were cultured on poly-L-lysine (PLL)-coated glass coverslips.^59^^,^^60^ Rats were obtained from the University Medical Center Göttingen, Germany, and sacrificed immediately after delivery. Both female and male rats were sacrificed for preparing the hippocampal cultures, in a 1:1 ratio. All procedures were performed in accordance with the regulatory standards and approved by the local authority, the Lower Saxony State Office for Consumer Protection and Food Safety (Niedersächsisches Landesamt für Verbraucherschutz und Lebensmittelsicherheit, document T.9-08). Cultures were maintained in 12-wells plates in Neurobasal-A medium (Gibco, Paisley, Scotland), pH 7.5, at 37°C, 5% CO_2_ in a humidified incubator.

### Method details

#### Overexpression of mEGFP tagged proteins of interest

For the expression of fluorescently tagged proteins, a monomeric variant of enhanced GFP (A206K mutant, mEGFP) was used as the fluorescent tag for the proteins of interest (POI). An empty pmEGFP-N1 vector gifted by Prof. Dr. Reinhard Jahn (Max Planck Institute for Biophysical Chemistry, Göttingen, Germany) was used for expression of a control protein mEGFP. Primary hippocampal neurons, at 4–5 days *in vitro* (DIV), were transfected using Lipofectamine 2000® reagent (Invitrogen, Carlsbad, CA, USA). Coverslips with neuronal cultures were transferred into 400 μl Dulbecco’s Modified Eagle Medium (DMEM, Thermo Fisher Scientific) supplemented with 10 mM MgCl_2_ and incubated for 30minat 37°C in 5% CO_2_. During this incubation, the transfection mix comprised of 2 μl of Lipofectamine 2000® reagent (Invitrogen, Carlsbad, CA, USA) and 1 μg of plasmid per well were combined in 50 μl of Opti-MEM® (Gibco, Paisley, Scotland). The lipofection mixture was added to the cells dropwise, and the neurons were further incubated for 30 minutes at 37°C in 5% CO_2_. Coverslips were then washed three times with DMEM supplemented with 10 mM MgCl_2_ and transferred back into their original conditioned Neurobasal medium.

#### Live immunolabelling of presynapses

To reveal active presynapses in culture, singlestep immunofluorescence was applied using pre-formed complexes of mouse monoclonal antibodies directed to the extracellular (luminal) domain of Synaptotagmin 1 (Synaptic Systems, 105311, Göttingen, Germany) and fluorescently labelled secondary nanobodies (NanoTag, N1202-Ab635P-S, Göttingen, Germany). The used concentrations for primary antibodies and secondary nanobodies were 15 nM and 30 nM, respectively (1:2 molar ratio). Antibodies and nanobodies were initially mixed in 20 μl of PBS and left for 30 minutes at RT, for complex formation. Thereafter, the complex was added directly to the neuronal medium and incubated for 1 hour at 37°C in 5% CO_2_. Thistype of functional immunolabelling of active presynapses was only performed for FRAP experiments on stubby spines (Figures 2A and S8–S14).

#### Fluorescent recovery after photobleaching (FRAP)

FRAP experiments were performed on the primary neurons 7 days after lipofection (i.e., at DIV11). Cultures were imaged using an inverted TCS SP5 laser scanning confocal microscope (Leica, Wetzlar, Germany) equipped with an oil immersion HCX Plan Apochromat 100× N.A. 1.40 objective. The mEGFP-tagged proteins and the functional labelling of active synapses (luminal domain of Synaptotagmin 1) were exited using a 488 nm Argon laser and a 633 nm Helium-neon laser, respectively, and emission detected using photomultiplier tube detectors (PMT: R 9624, Hamamatsu Photonics). Transfected neurons on coverslips were mounted on a self-made coverslip holder and 500 μl of pre-warmed Tyrode’s solution (124 mM NaCl, 2.7 mM KCl, 10 mM Na_2_HPO_4_, 2 mM KH_2_PO_4_, pH 7.3) were added. The whole microscopy body was enclosed in a chamber maintained at 37°C during image acquisition. Neuronal structures of interest such as, dendritic shafts, mushroom and stubby spines were located manually. Mushroom spines were located based on their morphological characteristics, while stubby spines were revealed in synapses after detection of recycled Synaptotagmin 1 live staining, scanning areas of 15 μm using 128 × 128 pixels with 122 nm pixel size. For the actual FRAP recordings, a single region of interest was recorded for 76 frames using 25 nm pixel size and 128 × 128 pixel resolution.

The 76 frames were comprised of 4 pre-bleaching images as well as 3 groups of 24 post bleaching images each, using different acquisition intervals. The fluorescent intensities from the pre-bleaching images were averaged and used as a pre-bleaching reference while, the 72 post-bleaching images were recorded to track the fluorescent recovery. The region of interest was photobleached for 80 ms using three excitation lasers at 488 nm, 496 nm and 476 nm, with power intensities (reaching the sample) of 50 μW, 14 μW and 15 μW, respectively. Thereafter, the 3 groups of post bleaching frames were acquired sequentially. For the first, second and third group we used 0.5 s, 1 s and 2 s acquisition intervals, respectively. Furthermore, control recordings were acquired on separate spines and dendritic shaft regions using the same acquisition and time settings, but with no laser used for bleaching. The control recordings were employed to correct for photo bleaching that was induced from repetitive recordings during FRAP experiments.

The total number of cultures (N) and the total number of neurons (n) imaged in the FRAP experiments, for each protein, are as follows: Amphiphysin, N = 3, n = 17; α-Synuclein, N = 2, n = 18; Calmodulin, N = 3, n = 18; Doc2a, N = 3, n = 16; Dynamin, N = 3, n = 17; Endophilin, N = 2, n = 14; mEGFP, N = 3, n = 21. The numbers of FRAP curves that were successfully analysed for each individual compartment are indicated in the Figures S8–S14, for each protein.

#### Immunolabeling and imaging for overexpression assessment

Transfected neurons at DIV11 were fixed with prewarmed solution of 4% paraformaldehyde (PFA, Sigma Aldrich) for 30 minutes at RT. After removing the PFA and shortly rinsing with PBS, unreacted aldehyde groups were quenched using 0.1 M glycine in PBS for 15 minutes. Cells were blocked and permeabilized with 2% bovine serum albumin (BSA, Sigma Aldrich) and 0.1% Triton X-100 (Sigma Aldrich) in PBS for 30 minutes at RT and gently shaking. Afterwards, pre-formed complexes of primary antibodies (20 nM) and fluorescently labelled secondary nanobodies (50 nM) were used for immunolabelling the protein of interest. Simultaneously, 20 nM of FluoTag®-X2 anti-PSD95 - AbberiorStar580 (N3702-Ab580-L, NanoTag Biotechnologies, Göttingen, Germany) were used for direct labelling of PSD95. For references to antibody validation methods see Table S1. The probes were incubated with neurons for 1 hour at RT in blocking and permeabilization solution. Finally, samples were washed three times with PBS, for 10 minutes each, and mounted on glass slides using Mowiol mounting medium (3 μM Mowiol® 4-88, 2.6 M Glycerol and 96 μM Tris-HCl pH: 8.5 in d.d. H_2_O). Images from overexpressing neurons were acquired using an inverted epifluorescence Nikon Eclipse Ti-E microscope, (Nikon Instruments Inc.) equipped with an IXON X3897 camera (Andor), an HBO-100W lamp and a 20x air or 60x oil immersion objective (Figure S16). The illumination settings were configured using the NIS-Elements AR software, version 4.60.00 (Nikon Corporation), and kept constant for imaging samples of the same protein of interest.

### Quantification and statistical analysis

#### FRAP image analysis

Custom-written MATLAB (The MathWorks Inc, Natick, MA, USA) routines were used for the supervised analysis of data from FRAP experiments. All frames acquired during FRAP experiments were loaded and the region of interest (ROI) was determined automatically by detecting the drop in fluorescence intensity between the pre- and the first postbleaching frame. Afterwards, each frame was corrected for the background intensity by subtracting the average intensity of non-cellular neighbouring regions, and the FRAP curve was generated. In addition, frames from FRAP movies were corrected for bleaching induced by the repetitive image acquisition, by determining an average bleaching curve. These average bleaching curves were obtained for each protein and compartment of interest by acquiring at least three movies per compartment, applying identical acquisition settings, however, without applying the bleaching step. Thereafter, the obtained FRAP curve was divided by the bleaching curve for the same culture, and a single exponential equation was fitted to the corrected FRAP curve to derive the mobility parameters, providing the results of Figures 2 and S8–S14. All generated curves and kinetics parameters (taus and immobile fractions) were inspected by an experienced investigator, to avoid using results from unusual or badly fitted curves.

#### Overexpression analysis

The protein overexpression levels were measured after localizing and quantifying the fluorescent signal from immunolabeled proteins of interest in both mEGFP-positive and mEGFP-negative postsynapses (Figure S17). We immunolabelled the PSD95 with a specific fluorescent nanobody (FluoTag®-X2 anti-PSD95) to delineate regions of interest in which the signal from all other channels was analysed (POI-AbberiorSTAR 635p and POI-mEGFP). We applied an empirically-derived threshold, in an automatic fashion, to detect PSD-positive spots, which became our relevant regions of interest (ROIs) for this analysis. The intensity in the GFP channel was used to determine whether each ROI belonged to a control neuron or to one expressing the protein of interest. We then measured the intensity of the protein of interest immunostaining in these ROIs and compared the intensities between GFP-positive and GFP-negative ROIs to quantify the overexpression levels. The data are presented in Figures S1–S6. The overexpression levels for each protein were quantified using two independent cultures, with at least 163 mEGFP-positive and 1076 mEGFP-negative ROIs analysed for each protein.

#### Statistical analysis

Results from the FRAP analysis of each protein of interest as well as the population of individual experiments are presented in Figures S8–S14. All statistical analysis procedures took place in GraphPad Prism (v. 9.1.2) and are described in the legends of each individual figure. The normality of the data was examined using the Shapiro-Wilk test (Table S2) and all plotted data are shown as mean ± SEM or as boxplots, as indicated in the respective figure legends. Statistical comparisons were done by nonparametric Kruskal-Wallis tests followed by multiple comparisons with Dunn’s correction, as indicated in the figure legends. To investigate the relation between kinetics parameters and molecular weight we used 2-tail Spearman correlation tests with Bonferroni correction for multiple testing.

## Data Availability

• The datasets supporting the current study have not been deposited in a public repository because they are presented almost fully in the different figures and tables, but are available from the lead contact upon request. • All original code has been deposited at https://figshare.com/ and is publicly available as of the date of publication. DOI is indicated in the key resources table. • Any additional information required to reanalyze the data reported in this paper is available from the lead contact upon request.
