# Supplementary material for: Trafficking proteins show limited differences in mobility across different postsynaptic spines
Source: iScience. 2023 Jan 13;26(2):105971. doi: 10.1016/j.isci.2023.105971 (PMC9883188; doi:10.1016/j.isci.2023.105971)
Supplement: Document S1. Figures S1–S17 and Tables S1 and S2 [file mmc1.pdf]

iScience, Volume *26*

Supplemental information

Trafficking proteins show limited differences in mobility across different postsynaptic spines

Nikolaos Mougios, Felipe Opazo, Silvio O. Rizzoli, and Sofiia Reshetniak

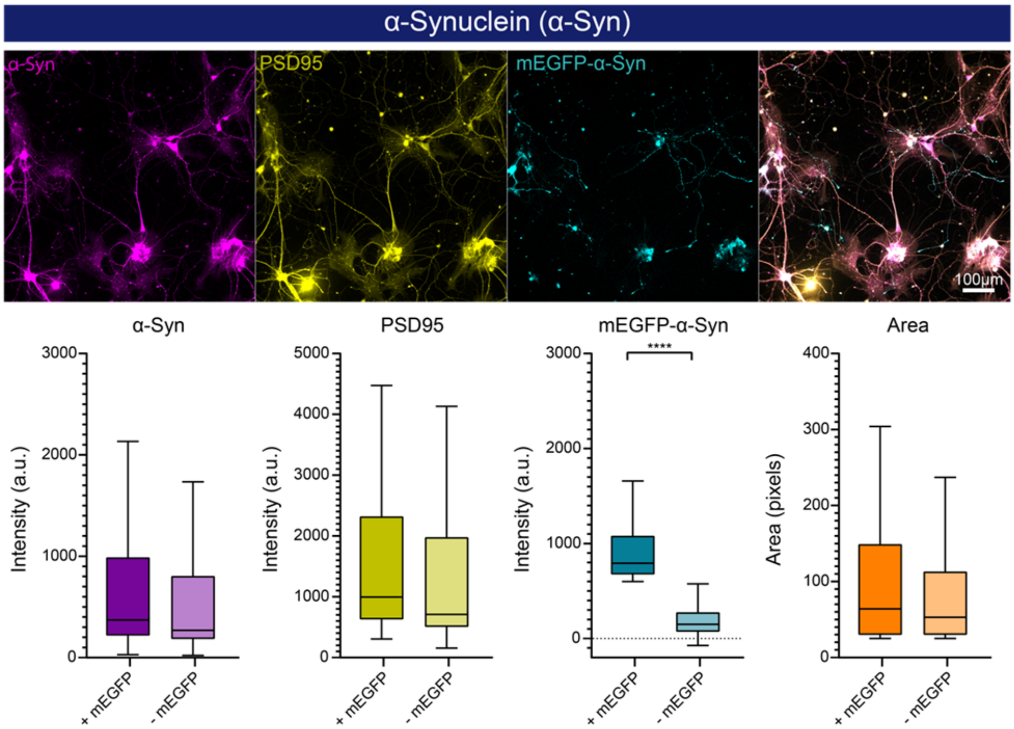

Figure S1: Overexpression analysis of α-synuclein tagged with mEGFP. Related to Figures 1-2. **Top:** Representative images from neuronal cultures expressing mEGFP-tagged α-synuclein (α-Syn). The α-synuclein immunolabelling is shown in magenta, with PSD95 in yellow and the mEGFP signal in cyan. Scale bar: 100 µm. **Bottom**: Box plots of fluorescence intensities from immunolabelled α-synuclein, PSD95, mEGFP and synapse area (based on immunolabeling of the protein of interest) in neurons with and without protein overexpression in mEGFP positive and mEGFP negative regions. Box plots are comprised of the median (middle line), the 25th and 75th percentile (box edges), and the min and max values (error bars). Asterisks represent statistical significance of $p < 0.0001$ after Kruskal-Wallis tests followed by Dunn's multiple testing procedure. See Figure S16 for higher zoom-in images.

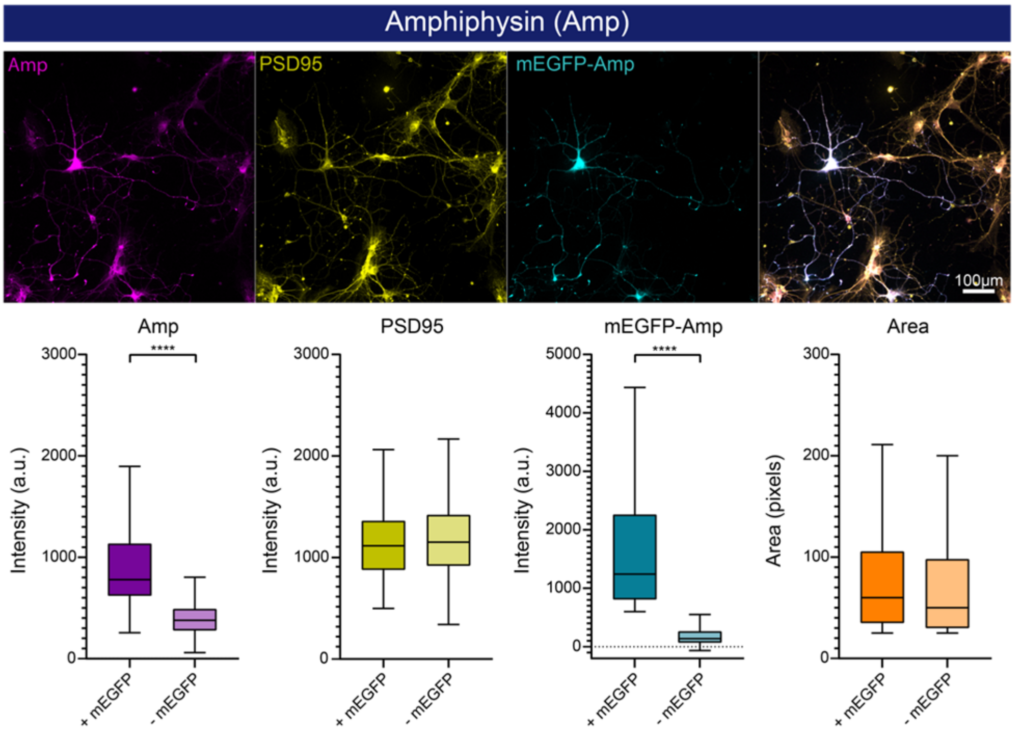

Figure S2: Overexpression analysis of amphiphysin tagged with mEGFP. Related to Figures 1-2. **Top**: Representative images from neuronal cultures expressing mEGFP-tagged amphiphysin (Amp). The amphiphysin immunolabelling is shown in magenta, with PSD95 in yellow and the mEGFP signal in cyan. Scale bar: 100 µm. **Bottom**: Box plots of fluorescence intensities from immunolabelled amphiphysin, PSD95, mEGFP and synapse area (based on immunolabeling of the protein of interest) in neurons with and without protein overexpression in mEGFP positive and mEGFP negative regions. Box plots are comprised of the median (middle line), the 25th and 75th percentile (box edges), and the min and max values (error bars). Asterisks represent statistical significance of $p < 0.0001$ after Kruskal-Wallis tests followed by Dunn's multiple testing procedure. See Figure S16 for higher zoom-in images.

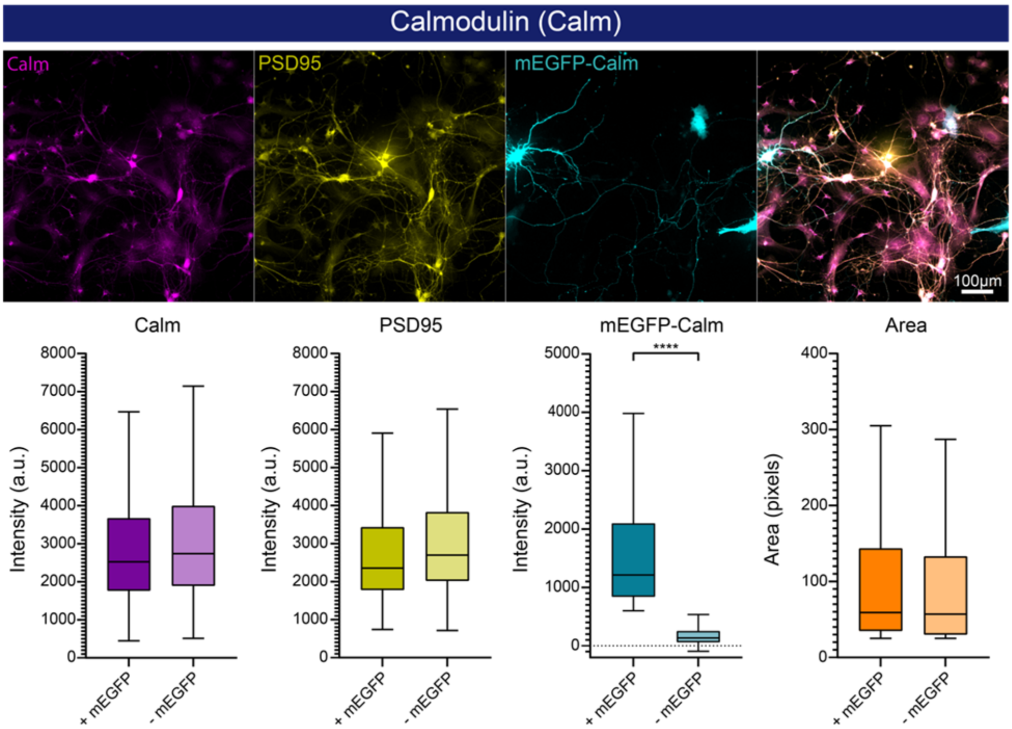

Figure S3: Overexpression analysis of calmodulin 1 tagged with mEGFP. Related to Figures 1-2. **Top:** Representative images from neuronal cultures expressing mEGFP-tagged calmodulin 1 (Calm)**.** The calmodulin 1 immunolabelling is shown in magenta, with PSD95 in yellow and the mEGFP signal in cyan. Scale bar: 100 µm. **Bottom**: Box plots of fluorescence intensities from immunolabelled calmodulin 1, PSD95, mEGFP and synapse area (based on immunolabeling of the protein of interest) in neurons with and without protein overexpression in mEGFP positive and mEGFP negative regions. Box plots are comprised of the median (middle line), the 25th and 75th percentile (box edges), and the min and max values (error bars). Asterisks represent statistical significance of $p < 0.0001$ after Kruskal-Wallis tests followed by Dunn's multiple testing procedure. See Figure S16 for higher zoom-in images.

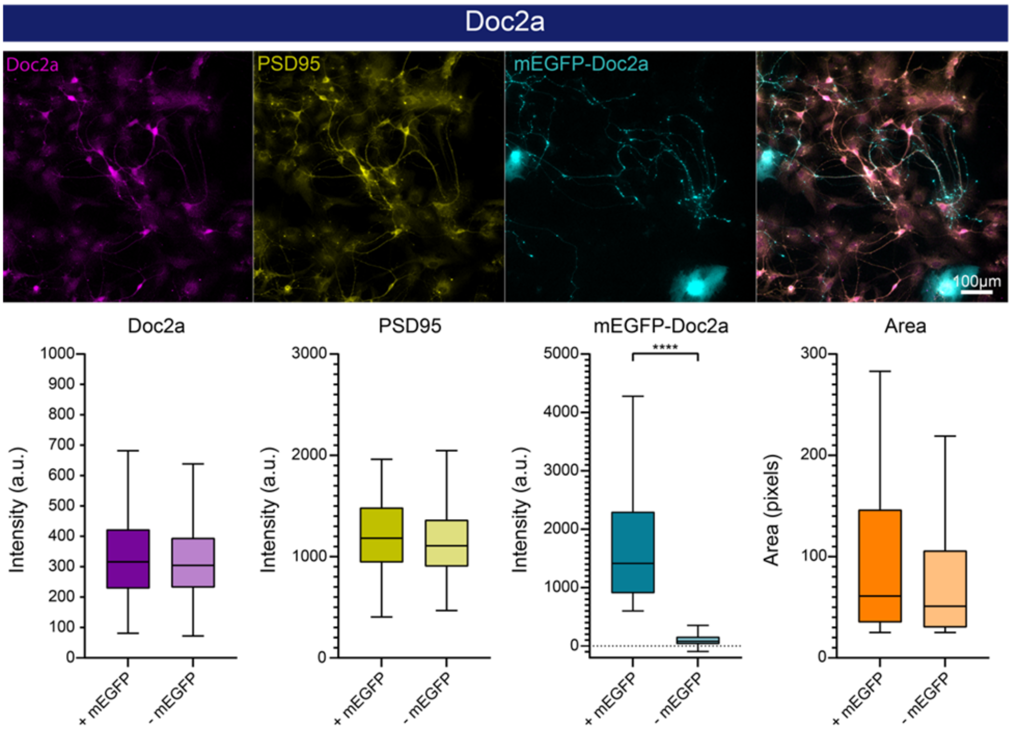

Figure S4: Overexpression analysis of doc2a tagged with mEGFP. Related to Figures 1-2. **Top**: Representative images from neuronal cultures expressing mEGFP-tagged doc2a**.** The doc2a immunolabelling is shown in magenta, with PSD95 in yellow and the mEGFP signal in cyan. Scale bar: 100 µm. **Bottom**: Box plots of fluorescence intensities from immunolabelled doc2a, PSD95, mEGFP and synapse area (based on immunolabeling of the protein of interest) in neurons with and without protein overexpression in mEGFP positive and mEGFP negative regions. Box plots are comprised of the median (middle line), the 25th and 75th percentile (box edges), and the min and max values (error bars). Asterisks represent statistical significance of $p < 0.0001$ after Kruskal-Wallis tests followed by Dunn's multiple testing procedure. See Figure S16 for higher zoom-in images.

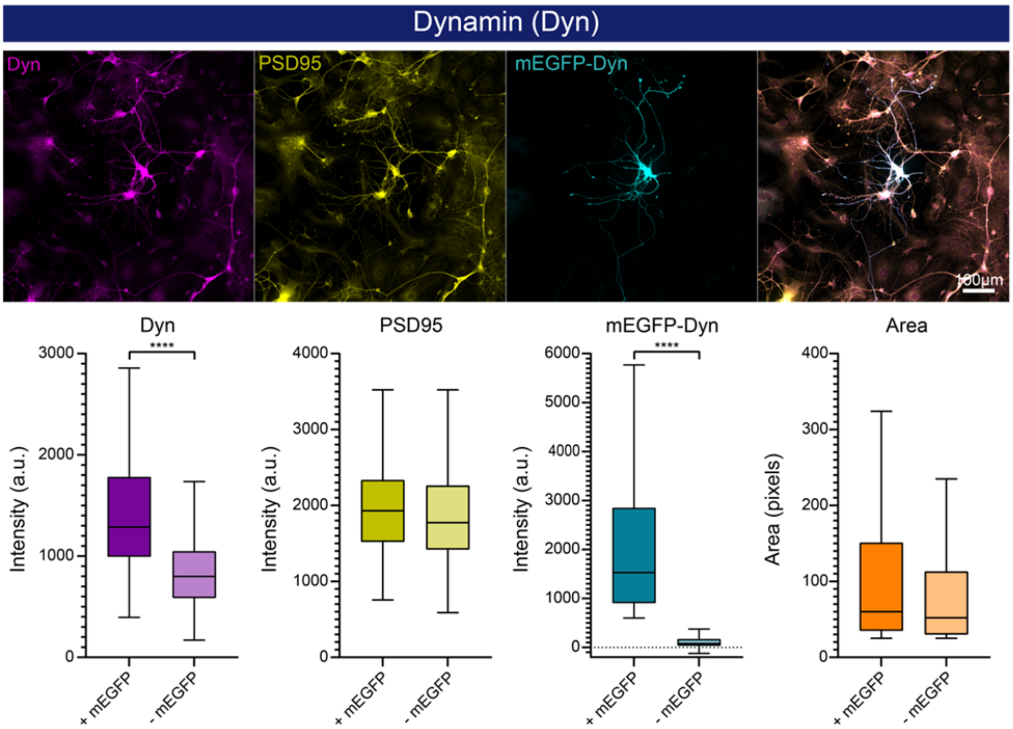

Figure S5: Overexpression analysis of dynamin 1 tagged with mEGFP. Related to Figures 1-2. **Top**: Representative images from neuronal cultures expressing mEGFP-tagged dynamin 1 (Dyn). The dynamin 1 immunolabelling is shown in magenta, with PSD95 in yellow and the mEGFP signal in cyan. Scale bar: 100 µm. **Bottom**: Box plots of fluorescence intensities from immunolabelled dynamin 1, PSD95, mEGFP and synapse area (based on immunolabeling of the protein of interest) in neurons with and without protein overexpression in mEGFP positive and mEGFP negative regions. Box plots are comprised of the median (middle line), the 25th and 75th percentile (box edges), and the min and max values (error bars). Asterisks represent statistical significance of $p < 0.0001$ after Kruskal-Wallis tests followed by Dunn's multiple testing procedure. See Figure S16 for higher zoom-in images.

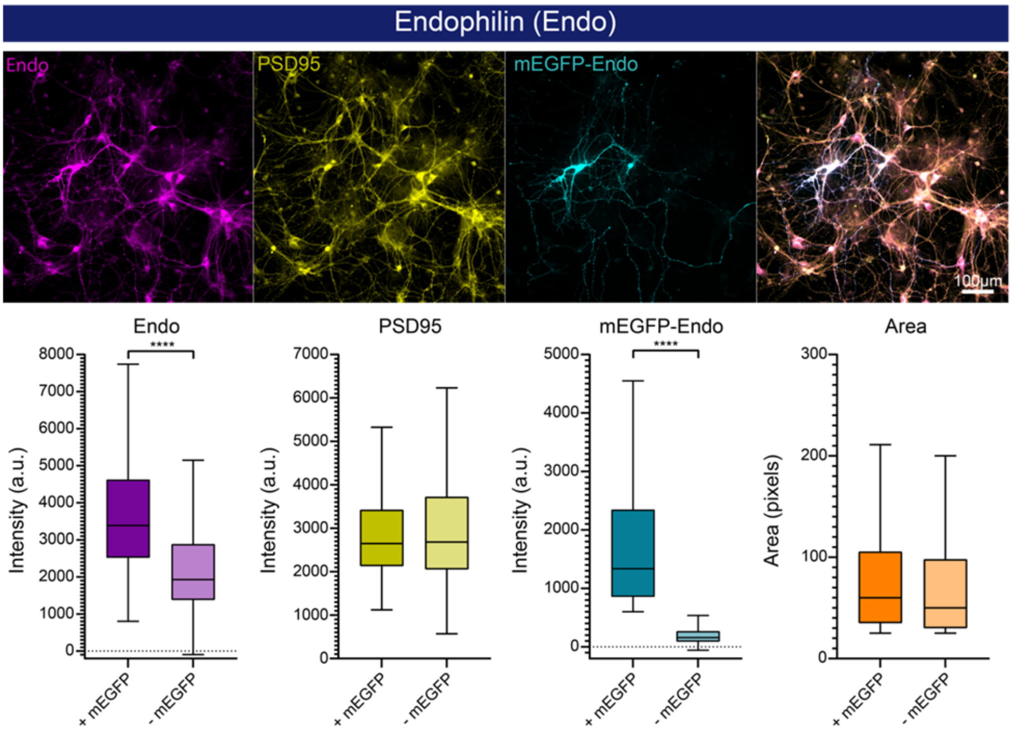

Figure S6: Overexpression analysis of endophilin A1 tagged with mEGFP. Related to Figures 1-2. **Top**: Representative images from neuronal cultures expressing mEGFP-tagged endophilin A1 (Endo). The endophilin A1 immunolabelling is shown in magenta, with PSD95 in yellow and the mEGFP signal in cyan. Scale bar: 100 µm. **Bottom**: Box plots of fluorescence intensities from immunolabelled endophilin A1, PSD95, mEGFP and synapse area (based on immunolabeling of the protein of interest) in neurons with and without protein overexpression in mEGFP positive and mEGFP negative regions. Box plots are comprised of the median (middle line), the 25th and 75th percentile (box edges), and the min and max values (error bars). Asterisks represent statistical significance of $p < 0.0001$ after Kruskal-Wallis tests followed by Dunn's multiple testing procedure. See Figure S16 for higher zoom-in images.

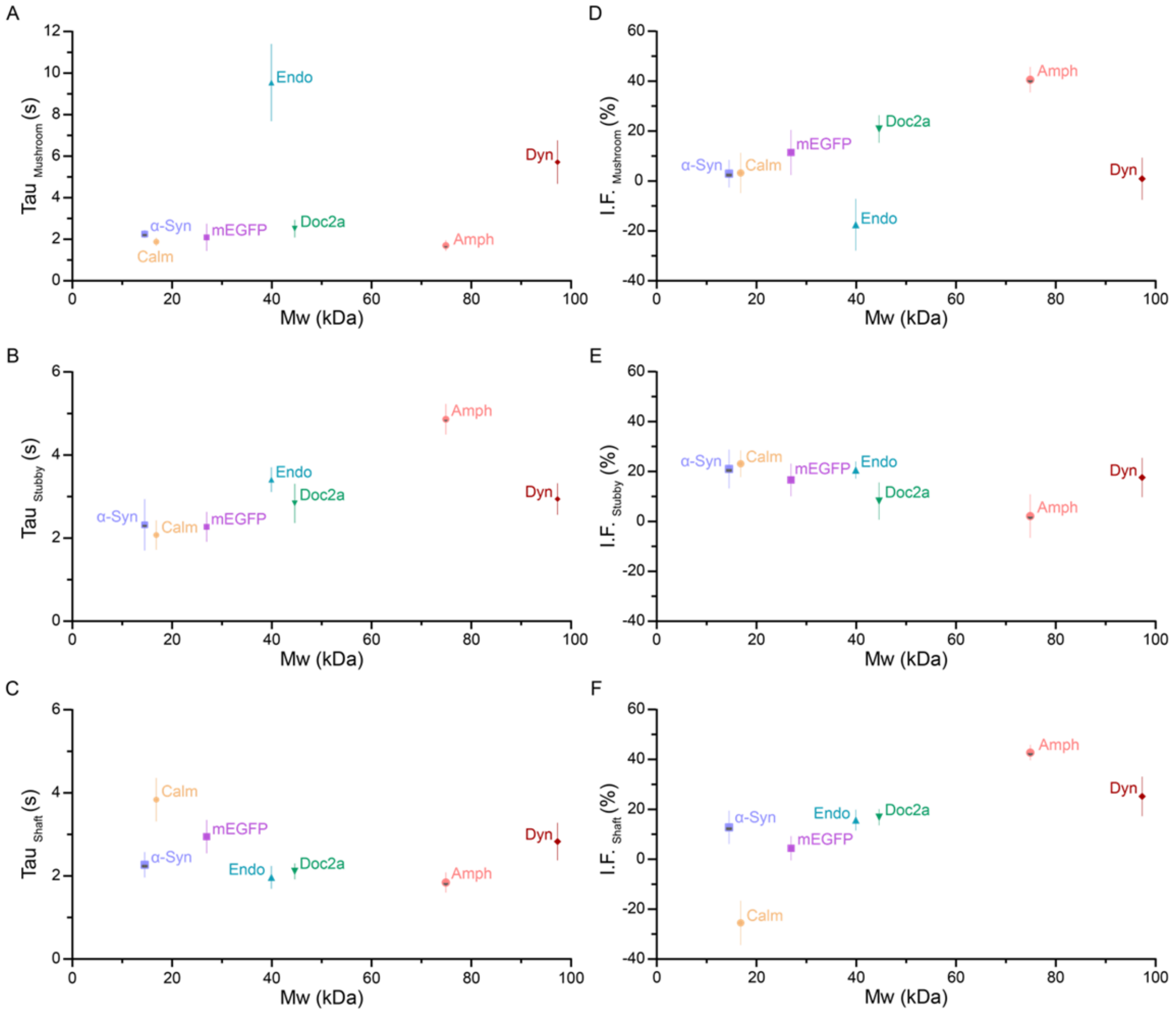

Figure S7: Scatter plots comparing molecular weight (Mw) with time constants (Tau) and immobile fractions (I.F.) from all analyzed proteins in mushroom spines (A and D), stubby spines (B and E) and dendritic shafts (C and F). Related to Figures 1-2. Symbols indicate mean ± SEM. No significant correlations were found, using two-tailed Spearman correlation tests and a Bonferroni correction for multiple testing. For Tau $P_{Mushroom}$ = 1, P_{Shaft} = 1, P_{Stubby} = 0.27. For I.F. $P_{Mushroom}$ = 1, P_{Shaft} = 0.07, P_{Stubby} = 0.33.

α-Synuclein							
Mw (kDa)	Category	Tau $_{Mushroom}$ (s)	Tau $_{Stubby}$ (s)	Tau $_{Shaft}$ (s)	I.F. $_{Mushroom}$ (%)	I.F. $_{Stubby}$ (%)	I.F. $_{Shaft}$ (%)
14,5	vesicle tethering	2,25 ± 0,19	2,32 ± 0,62	2,27 ± 0,31	2,95 ± 5,57	21,02 ± 7,78	12,80 ± 6,67

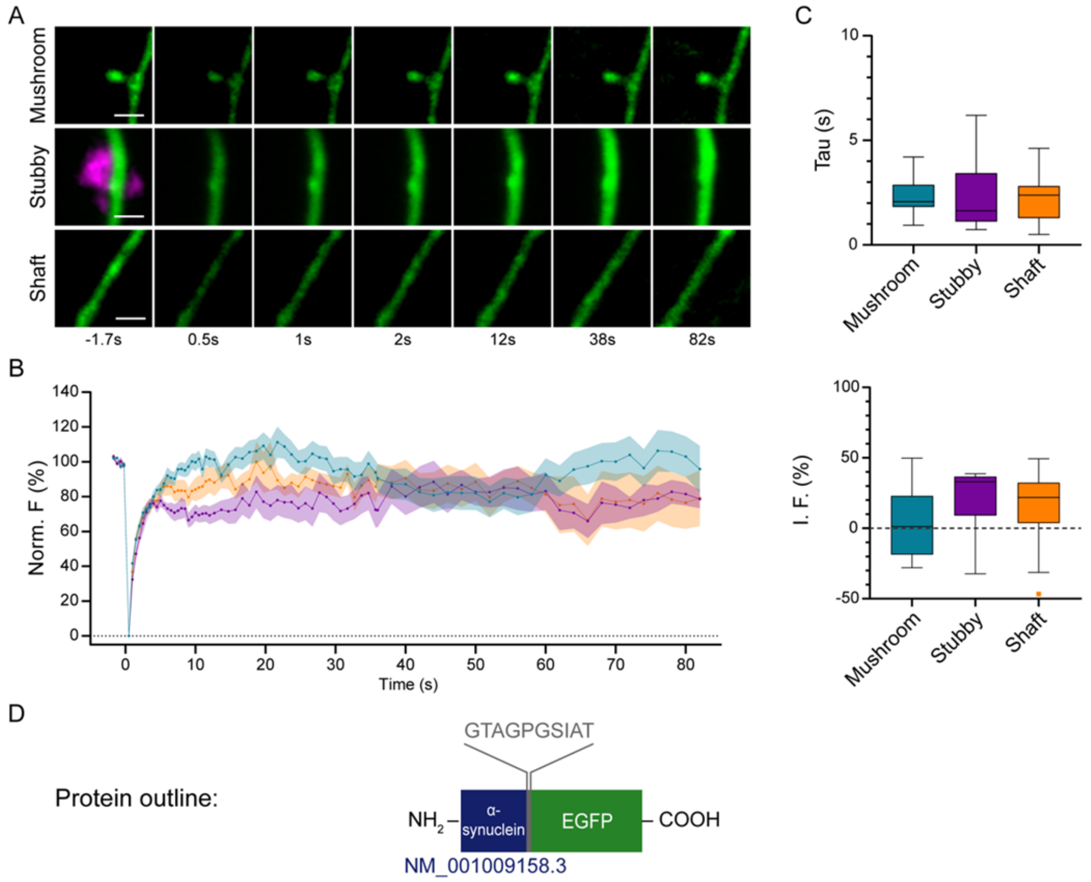

Figure S8: Summary of results from FRAP experiments of α-synuclein tagged with mEGFP. Related to Figure 2.

Top: Summary of α-synuclein characteristics, from left to right: molecular weight (Mw), category, mean time constant in mushroom spines ± SEM ($Tau_{Mushroom}$), mean time constant in stubby spines ± SEM (Tau_{Stubby}), mean time constant in dendritic shafts ± SEM (Tau_{Shaft}), mean immobile fraction in mushroom spines ± SEM ($I.F._{Mushroom}$), mean immobile fraction in stubby spines ± SEM ($I.F._{Stubby}$), mean immobile fraction in dendritic shafts ± SEM ($I.F._{Shaft}$). **(A)** Representative frames from mushroom spine (top), stubby spine (middle) and dendritic shaft (bottom), during FRAP experiment. The first was acquired 1.7 s before bleaching and the fluorescence recovery was monitored for 82 s in total, starting at 0.5 s after bleaching. Presynapse is shown in magenta. Scale bar: 1 µm. **(B)** FRAP curves from mushroom spines (green), stubby spines (purple) and dendritic shafts (orange). The points on the curve show the mean normalized fluorescent intensity and the shaded area indicates the ± SEM. **(C)** Box plots of time constants (top) and immobile fractions (bottom) in mushroom spines (green), stubby spines (purple) and dendritic shafts (orange). Box plots are comprised of the median (middle line), the 25th and 75th percentile (box edges), and the min and max values (error bars). Asterisks indicate significant differences after Kruskal-Wallis tests followed by Dunn's multiple testing procedure. α = 0.05. $N_{Mushroom}$ = 18, N_{Stubby} = 9, N_{Shaft} = 16.
(D) Schematic representation of the tagged α-synuclein. The protein, linker and fluorescent tag size ratios remain constant. The reference number of the mRNA sequence is shown as retrieved from Reshetniak et al. (2020) [S1].

(continued) **Kinetics comparisons:**
Time constant in mushroom spines was significantly different from time constant in mushroom spines of Endophilin A1 (p = 0.0382).
Immobile fraction in mushroom spines was significantly different from immobile fraction in mushroom spines of Amphiphysin (p = 0.0141).
Time constant in stubby spines was significantly different from time constant in stubby spines of Amphiphysin (p = 0.0051).
Immobile fraction in stubby spines was not significantly different from immobile fraction in stubby spines of any other protein.
Time constant in dendritic shafts was not significantly different from time constant in dendritic shafts of any other proteins.
Immobile fraction in dendritic shafts was not significantly different from immobile fraction in dendritic shafts of any other protein.
Time constant in mushroom spines was significantly different from time constant in pre-synapse (p = 0.0004).
Time constant in stubby spines was significantly different from time constant in pre-synapse (p = 0.0024).
Time constant in dendritic shaft was significantly different from time constant in pre-synapse (p = 0.0003).
Immobile fraction in mushroom spines was significantly different from immobile fraction in pre-synapse (p = 0.0237).
Immobile fraction in stubby spines was not significantly different from immobile fraction in any other neuronal compartment.
Immobile fraction in dendritic shaft was not significantly different from immobile fraction in any other neuronal compartment.
For statistics, the Kruskal-Wallis test with multiple comparisons and Dunn's correction was applied.

Amphiphysin							
Mw (kDa)	Category	Tau $_{Mushroom}$ (s)	Tau $_{Stubby}$ (s)	Tau $_{Shaft}$ (s)	I.F. $_{Mushroom}$ (%)	I.F. $_{Stubby}$ (%)	I.F. $_{Shaft}$ (%)
74,88	endocytic	1,70 ± 0,26	4,86 ± 0,37	1,84 ± 0,24	40,57 ± 5,13	2,14 ± 8,73	42,73 ± 3,10

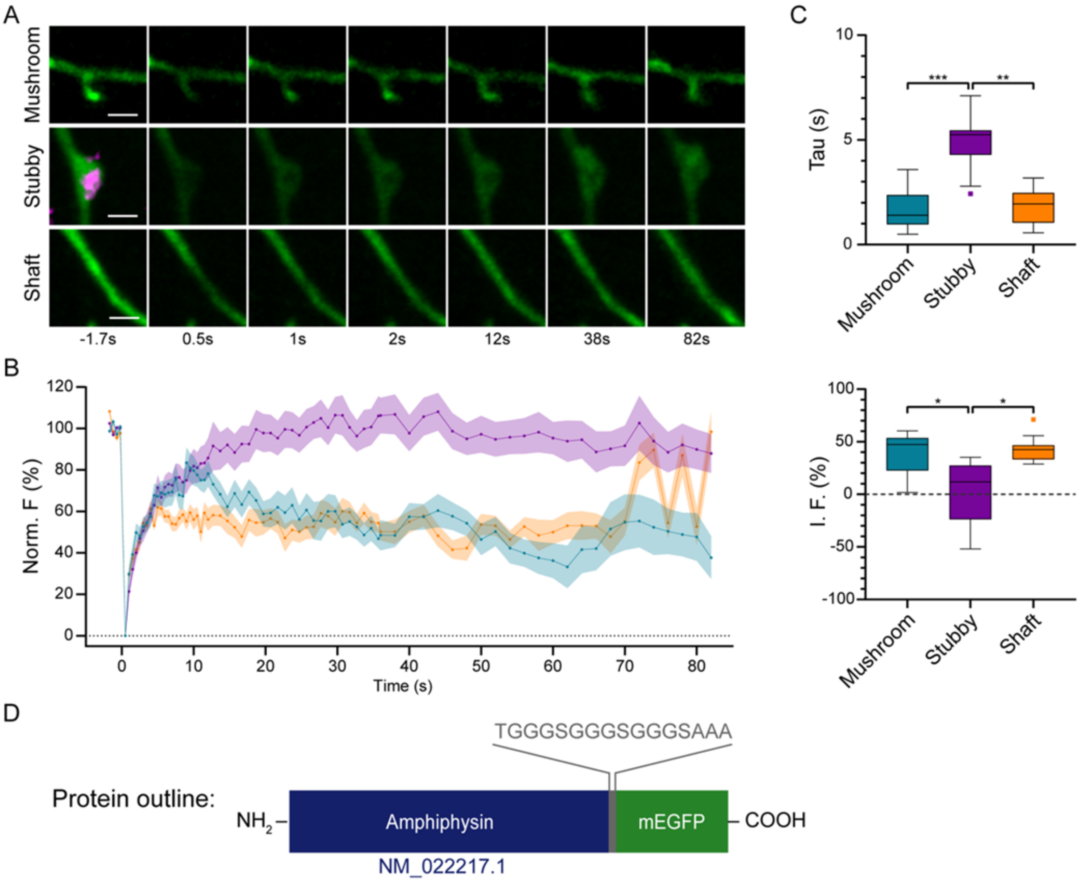

Figure S9: Summary of results from FRAP experiments of amphiphysin tagged with mEGFP. Related to Figure 2.

Top: Summary of amphiphysin characteristics, from left to right: molecular weight (Mw), category, mean time constant in mushroom spines ± SEM ($Tau_{Mushroom}$), mean time constant in stubby spines ± SEM (Tau_{Stubby}), mean time constant in dendritic shafts ± SEM (Tau_{Shaft}), mean immobile fraction in mushroom spines ± SEM ($I.F._{Mushroom}$), mean immobile fraction in stubby spines ± SEM ($I.F._{Stubby}$), mean immobile fraction in dendritic shafts ± SEM ($I.F._{Shaft}$). **(A)** Representative frames from mushroom spine (top), stubby spine (middle) and dendritic shaft (bottom), during FRAP experiment. The first was acquired 1.7 s before bleaching and the fluorescence recovery was monitored for 82 s in total, starting at 0.5 s after bleaching. Presynapse is shown in magenta. Scale bar: 1 μm. **(B)** FRAP curves from mushroom spines (green), stubby spines (purple) and dendritic shafts (orange). The points on the curve show the mean normalized fluorescent intensity and the shaded area indicates the ± SEM. **(C)** Box plots of time constants (top) and immobile fractions (bottom) in mushroom spines (green), stubby spines (purple) and dendritic shafts (orange). Box plots are comprised of the median (middle line), the 25th and 75th percentile (box edges), and the min and max values (error bars). Asterisks indicate significant differences after Kruskal-Wallis tests followed by Dunn's multiple testing procedure. α = 0.05. $N_{Mushroom}$ = 13, N_{Stubby} = 12, N_{Shaft} = 14. **(D)** Schematic representation of the tagged amphiphysin. The protein, linker and fluorescent tag size ratios remain constant. The reference number of the mRNA sequence is shown as retrieved from Reshetniak et al. (2020) [S1].

(continued) **Kinetics comparisons:**
Time constant in mushroom spines was significantly different from time constant in mushroom spines of Dynamin 1 ($p = 0.0067$) and Endophilin A1 ($p = 0.0006$).
Immobile fraction in mushroom spines was significantly different from immobile fraction in mushroom spines of α-Synuclein ($p = 0.0141$), Calmodulin 1 ($p = 0.0295$), Dynamin 1 ($p = 0.0202$) and Endophilin A1 ($p = 0.0011$).
Time constant in stubby spines was significantly different from time constant in stubby spines of α-Synuclein ($p = 0.0051$), Calmodulin 1 ($p = 0.0011$) and mEGFP ($p = 0.0027$).
Immobile fraction in stubby spines was not significantly different from immobile fraction in stubby spines of any other protein.
Time constant in dendritic shafts was not significantly different from time constant in dendritic shafts of any other proteins.
Immobile fraction in dendritic shafts was significantly different from immobile fraction in dendritic shafts of Calmodulin 1 ($p = < 0.0001$) and mEGFP ($p = 0.0011$).
Time constant in mushroom spines was significantly different from time constant in stubby spines ($p = 0.0009$) and pre-synapse ($p < 0.0001$).
Time constant in stubby spines was significantly different from time constant in mushroom spines ($p = 0.0009$) and dendritic shafts ($p = 0.0016$).
Time constant in dendritic shaft was significantly different from time constant in stubby spines ($p = 0.0016$) and pre-synapse ($p < 0.0001$).
Immobile fraction in mushroom spines was significantly different from immobile fraction in stubby spines ($p = 0.0145$).
Immobile fraction in stubby spines was significantly different from immobile fraction in mushroom spines ($p = 0.0145$) and dendritic shafts ($p = 0.0103$).
Immobile fraction in dendritic shaft was significantly different from immobile fraction in stubby spines ($p = 0.0103$).
For statistics, the Kruskal-Wallis test with multiple comparisons and Dunn's correction was applied.

Calmodulin							
Mw (kDa)	Category	$Tau_{Mushroom}$ (s)	Tau_{Stubby} (s)	Tau_{Shaft} (s)	$I.F._{Mushroom}$ (%)	$I.F._{Stubby}$ (%)	$I.F._{Shaft}$ (%)
16,84	calcium sensor	1,88 ± 0,20	2,07 ± 0,35	3,84 ± 0,52	3,23 ± 8,08	23,11 ± 5,35	-25,49 ± 8,89

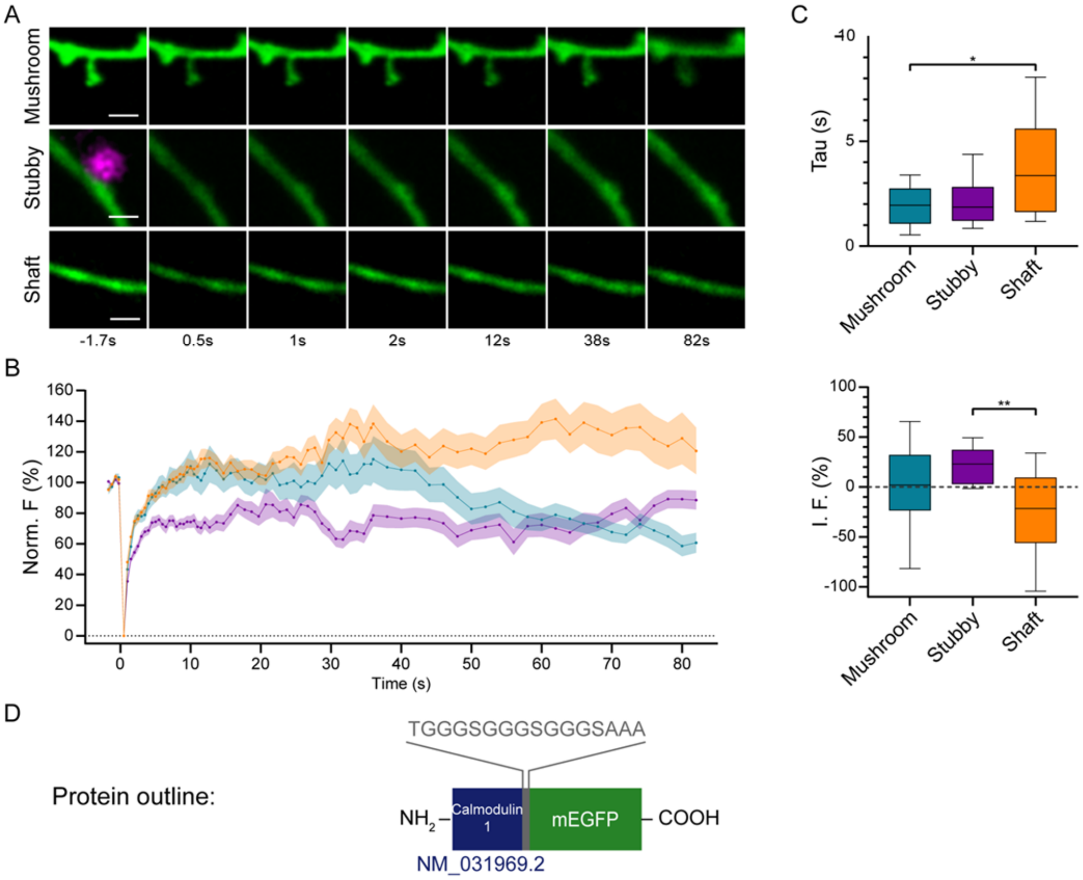

Figure S10: Summary of results from FRAP experiments of calmodulin 1 tagged with mEGFP. Related to Figure 2.
Top: Summary of calmodulin 1 characteristics, from left to right: molecular weight (Mw), category, mean time constant in mushroom spines ± SEM ($Tau_{Mushroom}$), mean time constant in stubby spines ± SEM (Tau_{Stubby}), mean time constant in dendritic shafts ± SEM (Tau_{Shaft}), mean immobile fraction in mushroom spines ± SEM ($I.F._{Mushroom}$), mean immobile fraction in stubby spines ± SEM ($I.F._{Stubby}$), mean immobile fraction in dendritic shafts ± SEM ($I.F._{Shaft}$). **(A)** Representative frames from mushroom spine (top), stubby spine (middle) and dendritic shaft (bottom), during FRAP experiment. The first was acquired 1.7 s before bleaching and the fluorescence recovery was monitored for 82 s in total, starting at 0.5 s after bleaching. Presynapse is shown in magenta. Scale bar: 1 µm. **(B)** FRAP curves from mushroom spines (green), stubby spines (purple) and dendritic shafts (orange). The points on the curve show the mean normalized fluorescent intensity and the shaded area indicates the ± SEM. **(C)** Box plots of time constants (top) and immobile fractions (bottom) in mushroom spines (green), stubby spines (purple) and dendritic shafts (orange). Box plots are comprised of the median (middle line), the 25th and 75th percentile (box edges), and the min and max values (error bars). Asterisks indicate significant differences after Kruskal-Wallis tests followed by Dunn's multiple testing procedure. α = 0.05. $N_{Mushroom}$ = 20, N_{Stubby} = 11, N_{Shaft} = 20. **(D)** Schematic representation of the tagged calmodulin 1. The protein, linker and fluorescent tag size ratios remain constant. The reference number of the mRNA sequence is shown as retrieved from Reshetniak et al. (2020) [S1].

(continued) **Kinetics comparisons:**
Time constant in mushroom spines was significantly different from time constant in mushroom spines of Dynamin 1 ($p = 0.0116$) and Endophilin A1 ($p = 0.0011$).
Immobile fraction in mushroom spines was significantly different from immobile fraction in mushroom spines of Amphiphysin ($p = 0.0295$).
Time constant in stubby spines was significantly different from time constant in stubby spines of Amphiphysin ($p = 0.0011$).
Immobile fraction in stubby spines was not significantly different from immobile fraction in stubby spines of any other protein.
Time constant in dendritic shafts was not significantly different from time constant in dendritic shafts of any other proteins.
Immobile fraction in dendritic shafts was significantly different from immobile fraction in dendritic shafts of Amphiphysin ($p < 0.0001$) and Dynamin ($p = 0.0019$).
Time constant in mushroom spines was significantly different from time constant in dendritic shafts ($p = 0.0115$) and pre-synapse ($p = 0.0221$).
Time constant in stubby spines was not significantly different from time constant in any other neuronal compartment.
Time constant in dendritic shaft was significantly different from time constant in mushroom spines ($p = 0.0115$).
Immobile fraction in mushroom spines was not significantly different from immobile fractions in any other neuronal compartment.
Immobile fraction in stubby spines was significantly different from immobile fraction in dendritic shaft ($p = 0.0026$).
Immobile fraction in dendritic shaft was significantly different from immobile fraction in stubby spines ($p = 0.0026$), pre-synapse ($p = 0.0006$) and axon ($p = 0.0149$).
For statistics, the Kruskal-Wallis test with multiple comparisons and Dunn's correction was applied.

Doc2a							
Mw (kDa)	Category	Tau $_{Mushroom}$ (s)	Tau $_{Stubby}$ (s)	Tau $_{Shaft}$ (s)	I.F. $_{Mushroom}$ (%)	I.F. $_{Stubby}$ (%)	I.F. $_{Shaft}$ (%)
44,59	calcium sensor	2,51 ± 0,42	2,84 ± 0,47	2,11 ± 0,19	20,82 ± 5,53	8,13 ± 7,47	16,83 ± 3,26

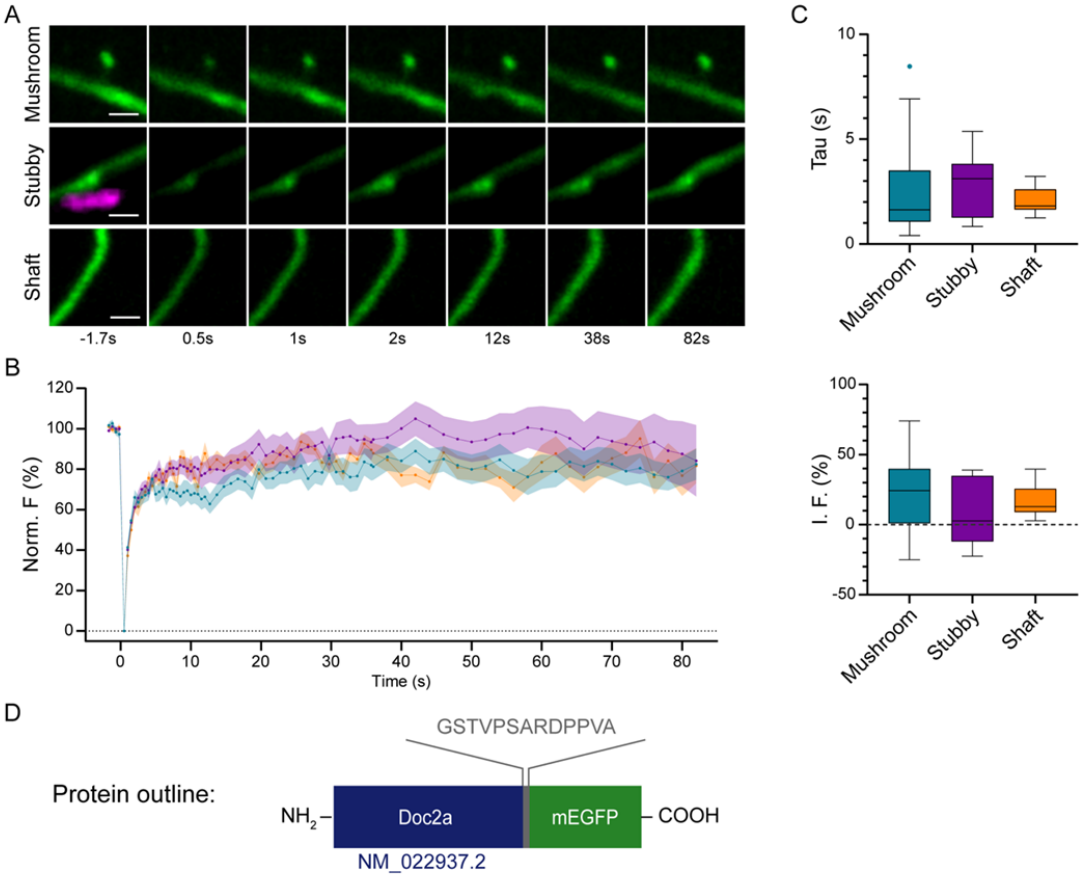

Figure S11: Summary of results from FRAP experiments of doc2a tagged with mEGFP. Related to Figure 2.

Top: Summary of doc2a characteristics, from left to right: molecular weight (Mw), category, mean time constant in mushroom spines ± SEM ($Tau_{Mushroom}$), mean time constant in stubby spines ± SEM (Tau_{Stubby}), mean time constant in dendritic shafts ± SEM (Tau_{Shaft}), mean immobile fraction in mushroom spines ± SEM ($I.F._{Mushroom}$), mean immobile fraction in stubby spines ± SEM ($I.F._{Stubby}$), mean immobile fraction in dendritic shafts ± SEM ($I.F._{Shaft}$). **(A)** Representative frames from mushroom spine (top), stubby spine (middle) and dendritic shaft (bottom), during FRAP experiment. The first was acquired 1.7 s before bleaching and the fluorescence recovery was monitored for 82 s in total, starting at 0.5 s after bleaching. Presynapse is shown in magenta. Scale bar: 1 µm. **(B)** FRAP curves from mushroom spines (green), stubby spines (purple) and dendritic shafts (orange). The points on the curve show the mean normalized fluorescent intensity and the shaded area indicates the ± SEM. **(C)** Box plots of time constants (top) and immobile fractions (bottom) in mushroom spines (green), stubby spines (purple) and dendritic shafts (orange). Box plots are comprised of the median (middle line), the 25th and 75th percentile (box edges), and the min and max values (error bars). Asterisks indicate significant differences after Kruskal-Wallis tests followed by Dunn's multiple testing procedure. α = 0.05. $N_{Mushroom}$ = 25, N_{Stubby} = 10, N_{Shaft} = 11. **(D)** Schematic representation of the tagged doc2a. The protein, linker and fluorescent tag size ratios remain constant. The reference number of the mRNA sequence is shown as retrieved from Reshetniak et al. (2020) [S1].

(continued) **Kinetics comparisons:**
Time constant in mushroom spines was significantly different from time constant in mushroom spines of Dynamin 1 ($p = 0.0186$) and Endophilin A1 ($p = 0.0017$).
Immobile fraction in mushroom spines was not significantly different from immobile fraction in mushroom spines of any other protein.
Time constant in stubby spines was not significantly different from time constant in stubby spines of any other protein.
Immobile fraction in stubby spines was not significantly different from immobile fraction in stubby spines of any other protein.
Time constant in dendritic shafts was not significantly different from time constant in dendritic shafts of any other proteins.
Immobile fraction in dendritic shafts was not significantly different from immobile fraction in dendritic shafts of any other protein.
Time constant in mushroom spines was significantly different from time constant in pre-synapse ($p = 0.0379$).
Time constant in stubby spines was not significantly different from time constant in any other neuronal compartment.
Time constant in dendritic shaft was not significantly different from time constant in any other neuronal compartment.
Immobile fraction in mushroom spines was not significantly different from immobile fractions in any other neuronal compartment.
Immobile fraction in stubby spines was not significantly different from immobile fractions in any other neuronal compartment.
Immobile fraction in dendritic shaft was not significantly different from immobile fractions in any other neuronal compartment.
For statistics, the Kruskal-Wallis test with multiple comparisons and Dunn's correction was applied.

Dynamin							
Mw (kDa)	Category	Tau$_{Mushroom}$ (s)	Tau$_{Stubby}$ (s)	Tau$_{Shaft}$ (s)	I.F.$_{Mushroom}$ (%)	I.F.$_{Stubby}$ (%)	I.F.$_{Shaft}$ (%)
97,29	endocytic	5,71 ± 1,05	2,94 ± 0,38	2,83 ± 0,45	0,91 ± 8,48	17,58 ± 7,87	25,17 ± 7,93

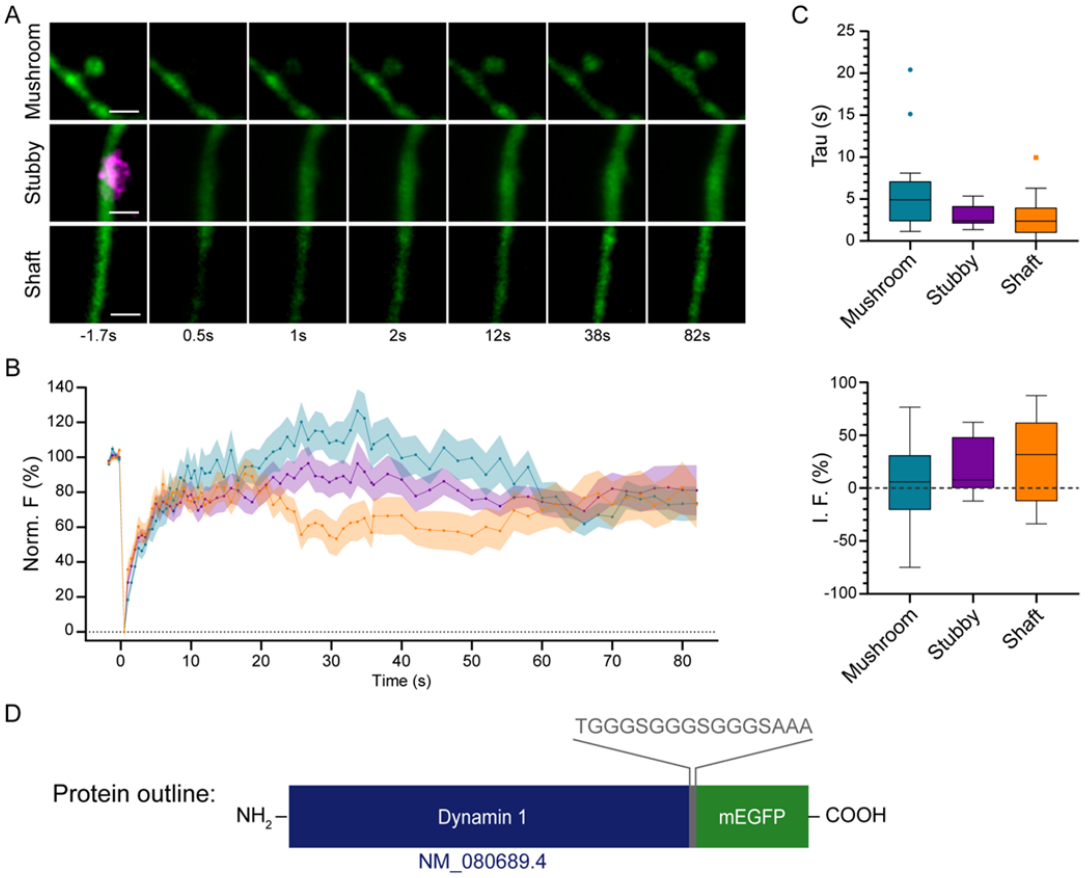

Figure S12: Summary of results from FRAP experiments of dynamin 1 tagged with mEGFP. Related to Figure 2.

Top: Summary of dynamin 1 characteristics, from left to right: molecular weight (Mw), category, mean time constant in mushroom spines ± SEM (Tau$_{Mushroom}$), mean time constant in stubby spines ± SEM (Tau$_{Stubby}$), mean time constant in dendritic shafts ± SEM (Tau$_{Shaft}$), mean immobile fraction in mushroom spines ± SEM (I.F.$_{Mushroom}$), mean immobile fraction in stubby spines ± SEM (I.F.$_{Stubby}$), mean immobile fraction in dendritic shafts ± SEM (I.F.$_{Shaft}$). **(A)** Representative frames from mushroom spine (top), stubby spine (middle) and dendritic shaft (bottom), during FRAP experiment. The first was acquired 1.7 s before bleaching and the fluorescence recovery was monitored for 82 s in total, starting at 0.5 s after bleaching. Presynapse is shown in magenta. Scale bar: 1 μm. **(B)** FRAP curves from mushroom spines (green), stubby spines (purple) and dendritic shafts (orange). The points on the curve show the mean normalized fluorescent intensity and the shaded area indicates the ± SEM. **(C)** Box plots of time constants (top) and immobile fractions (bottom) in mushroom spines (green), stubby spines (purple) and dendritic shafts (orange). Box plots are comprised of the median (middle line), the 25th and 75th percentile (box edges), and the min and max values (error bars). Asterisks indicate significant differences after Kruskal-Wallis tests followed by Dunn's multiple testing procedure. α = 0.05. $N_{Mushroom}$ = 20, N_{Stubby} = 11, N_{Shaft} = 25. **(D)** Schematic representation of the tagged dynamin. The protein, linker and fluorescent tag size ratios remain constant. The reference number of the mRNA sequence is shown as retrieved from Reshetniak et al. (2020) [S1].

(continued) **Kinetics comparisons:**
Time constant in mushroom spines was significantly different from time constant in mushroom spines of Amphiphysin ($p = 0.0067$), Calmodulin 1 ($p = 0.0116$), Doc2a ($p = 0.0186$) and mEGFP ($p < 0.0001$).
Immobile fraction in mushroom spines was significantly different from immobile fraction in mushroom spines of Amphiphysin ($p = 0.0202$).
Time constant in stubby spines was not significantly different from time constant in stubby spines of any other protein.
Immobile fraction in stubby spines was not significantly different from immobile fraction in stubby spines of any other protein.
Time constant in dendritic shafts was not significantly different from time constant in dendritic shafts of any other proteins.
Immobile fraction in dendritic shafts was significantly different from immobile fraction in dendritic shafts of Calmodulin 1 ($p = 0.0019$).
Time constant in mushroom spines was not significantly different from time constant in any other neuronal compartment.
Time constant in stubby spines was significantly different from time constant in pre-synapse ($p = 0.0011$).
Time constant in dendritic shaft was significantly different from time constant in pre-synapse ($p < 0.0001$).
Immobile fraction in mushroom spines was not significantly different from immobile fractions in any other neuronal compartment.
Immobile fraction in stubby spines was not significantly different from immobile fractions in any other neuronal compartment.
Immobile fraction in dendritic shaft was not significantly different from immobile fractions in any other neuronal compartment.
For statistics, the Kruskal-Wallis test with multiple comparisons and Dunn's correction was applied.

Endophilin							
Mw (kDa)	Category	Tau Mushroom (s)	Tau Stubby (s)	Tau Shaft (s)	I.F. Mushroom (%)	I.F. Stubby (%)	I.F. Shaft (%)
39,90	endocytic	9,54 ± 1,86	3,41 ± 0,30	1,97 ± 0,27	-17,52 ± 10,37	20,57 ± 3,46	15,71 ± 4,14

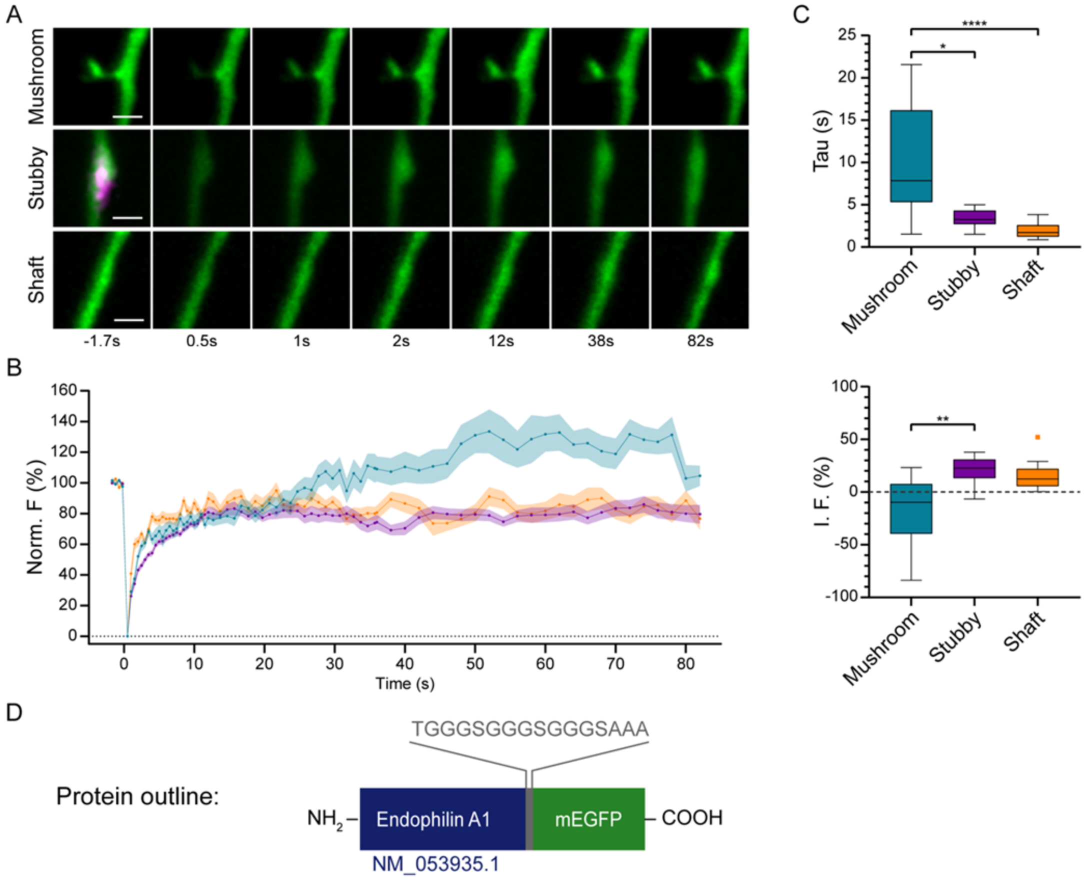

Figure S13: Summary of results from FRAP experiments of endophilin A1 tagged with mEGFP. Related to Figure 2.

Top: Summary of endophilin A1 characteristics, from left to right: molecular weight (Mw), category, mean time constant in mushroom spines ± SEM ($Tau_{Mushroom}$), mean time constant in stubby spines ± SEM (Tau_{Stubby}), mean time constant in dendritic shafts ± SEM (Tau_{Shaft}), mean immobile fraction in mushroom spines ± SEM ($I.F._{Mushroom}$), mean immobile fraction in stubby spines ± SEM ($I.F._{Stubby}$), mean immobile fraction in dendritic shafts ± SEM ($I.F._{Shaft}$). **(A)** Representative frames from mushroom spine (top), stubby spine (middle) and dendritic shaft (bottom), during FRAP experiment. The first was acquired 1.7 s before bleaching and the fluorescence recovery was monitored for 82 s in total, starting at 0.5 s after bleaching. Presynapse is shown in magenta. Scale bar: 1 μm. **(B)** FRAP curves from mushroom spines (green), stubby spines (purple) and dendritic shafts (orange). The points on the curve show the mean normalized fluorescent intensity and the shaded area indicates the ± SEM. **(C)** Box plots of time constants (top) and immobile fractions (bottom) in mushroom spines (green), stubby spines (purple) and dendritic shafts (orange). Box plots are comprised of the median (middle line), the 25th and 75th percentile (box edges), and the min and max values (error bars). Asterisks indicate significant differences after Kruskal-Wallis tests followed by Dunn's multiple testing procedure. α = 0.05. $N_{Mushroom}$ = 11, N_{Stubby} = 14, N_{Shaft} = 12. **(D)** Schematic representation of the tagged endophilin A1. The protein, linker and fluorescent tag size ratios remain constant. The reference number of the mRNA sequence is shown as retrieved from Reshetniak et al. (2020) [S1].

(continued) **Kinetics comparisons:**
Time constant in mushroom spines was significantly different from time constant in mushroom spines of α-Synuclein ($p = 0.0382$), Amphiphysin ($p = 0.0006$), Calmodulin 1 ($p = 0.0011$), Doc2a ($p = 0.0017$) and mEGFP (p = < 0.0001).
Immobile fraction in mushroom spines was significantly different from immobile fraction in mushroom spines of Amphiphysin ($p = 0.0011$).
Time constant in stubby spines was not significantly different from time constant in stubby spines of any other protein.
Immobile fraction in stubby spines was not significantly different from immobile fraction in stubby spines of any other protein.
Time constant in dendritic shafts was not significantly different from time constant in dendritic shafts of any other proteins.
Immobile fraction in dendritic shafts was not significantly different from immobile fraction in dendritic shafts of any other protein.
Time constant in mushroom spines was significantly different from time constant in stubby spines ($p = 0.0447$), dendritic shafts ($p < 0.0001$) and axon ($p < 0.0001$).
Time constant in stubby spines was significantly different from time constant in mushroom spines ($p = 0.0447$).
Time constant in dendritic shaft was significantly different from time constant in mushroom spines ($p < 0.0001$) and in pre-synapse ($p = 0.0002$).
Immobile fraction in mushroom spines was significantly different from immobile fraction in stubby spines (0.0032) and pre-synapse ($p = 0.0022$).
Immobile fraction in stubby spines was significantly different from immobile fraction in mushroom spines ($p = 0.0032$).
Immobile fraction in dendritic shaft was not significantly different from immobile fractions in any other neuronal compartment.
For statistics, the Kruskal-Wallis test with multiple comparisons and Dunn's correction was applied.

mEGFP							
Mw (kDa)	Category	Tau Mushroom (s)	Tau Stubby (s)	Tau Shaft (s)	I.F. Mushroom (%)	I.F. Stubby (%)	I.F. Shaft (%)
26,92	control	2,09 ± 0,66	2,27 ± 0,36	2,95 ± 0,40	11,45 ± 9,01	16,61 ± 6,56	4,39 ± 4,90

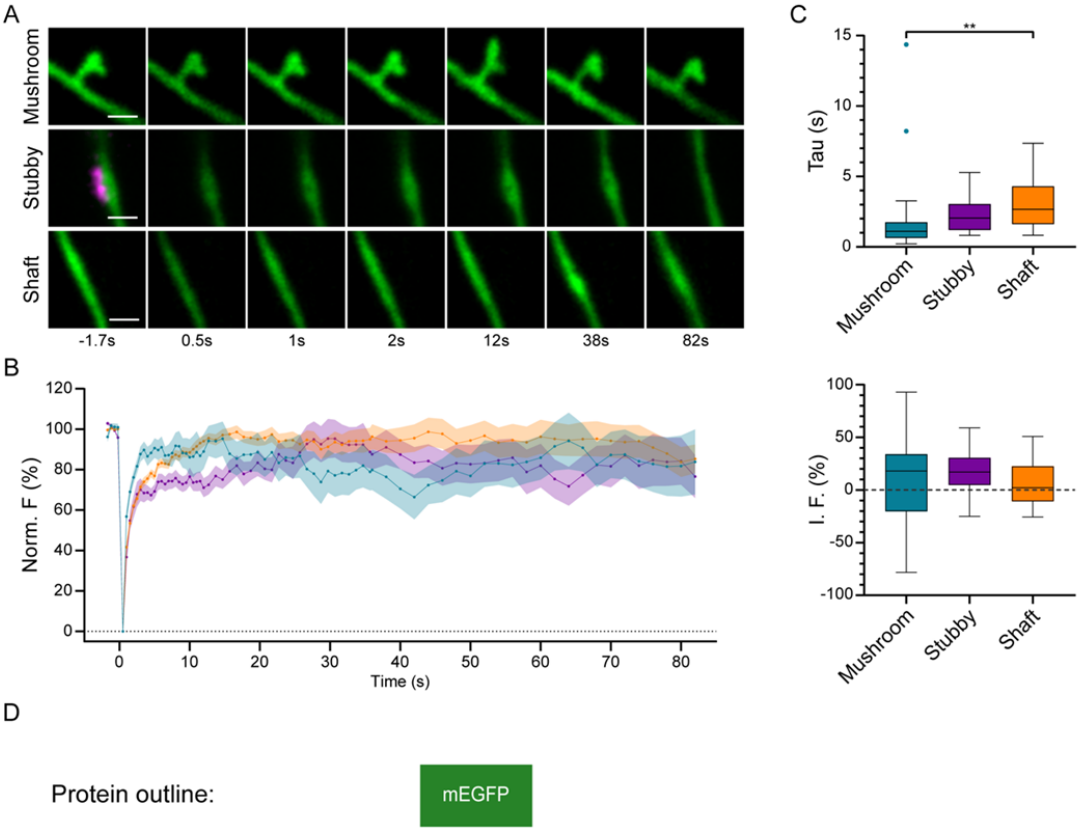

Figure S14: Summary of results from FRAP experiments of mEGFP. Related to Figure 2.
Top: Summary of mEGFP characteristics, from left to right: molecular weight (Mw), category, mean time constant in mushroom spines ± SEM ($Tau_{Mushroom}$), mean time constant in stubby spines ± SEM (Tau_{Stubby}), mean time constant in dendritic shafts ± SEM (Tau_{Shaft}), mean immobile fraction in mushroom spines ± SEM ($I.F._{Mushroom}$), mean immobile fraction in stubby spines ± SEM ($I.F._{Stubby}$), mean immobile fraction in dendritic shafts ± SEM ($I.F._{Shaft}$). **(A)** Representative frames from mushroom spine (top), stubby spine (middle) and dendritic shaft (bottom), during FRAP experiment. The first was acquired 1.7 s before bleaching and the fluorescence recovery was monitored for 82 s in total, starting at 0.5 s after bleaching. Presynapse is shown in magenta. Scale bar: 1 μm. **(B)** FRAP curves from mushroom spines (green), stubby spines (purple) and dendritic shafts (orange). The points on the curve show the mean normalized fluorescent intensity and the shaded area indicates the ± SEM. **(C)** Box plots of time constants (top) and immobile fractions (bottom) in mushroom spines (green), stubby spines (purple) and dendritic shafts (orange). Box plots are comprised of the median (middle line), the 25th and 75th percentile (box edges), and the min and max values (error bars). Asterisks indicate significant differences after Kruskal-Wallis tests followed by Dunn's multiple testing procedure. $\alpha = 0.05$. $N_{Mushroom} = 23$, $N_{Stubby} = 12$, $N_{Shaft} = 19$. **(D)** Schematic representation of the mEGFP. The protein, linker and fluorescent tag size ratios remain constant. The reference number of the mRNA sequence is shown as retrieved from Reshetniak et al. (2020) [S1].

(continued) **Kinetics comparisons:**
Time constant in mushroom spines was significantly different from time constant in mushroom spines of Dynamin 1 ($p = < 0.0001$) and Endophilin A1 ($p = < 0.0001$).
Immobile fraction in mushroom spines was not significantly different from immobile fraction in mushroom spines of any other protein.
Time constant in stubby spines was significantly different from time constant in stubby spines of Amphiphysin ($p = 0.0027$).
Immobile fraction in stubby spines was not significantly different from immobile fraction in stubby spines of any other protein.
Time constant in dendritic shafts was not significantly different from time constant in dendritic shafts of any other proteins.
Immobile fraction in dendritic shafts was significantly different from immobile fraction in dendritic shafts of Amphiphysin ($p = 0.0011$).
Time constant in mushroom spines was significantly different from time constant in dendritic shaft ($p = 0.0013$) and pre-synapse ($p = 0.0071$).
Time constant in stubby spines was not significantly different from time constants in any other neuronal compartment.
Time constant in dendritic shaft was significantly different from time constant in mushroom spines ($p = 0.0013$).
Immobile fraction in mushroom spines was not significantly different from immobile fractions in any other neuronal compartment.
Immobile fraction in stubby spines was not significantly different from immobile fractions in any other neuronal compartment.
Immobile fraction in dendritic shaft was significantly different from immobile fractions in axons ($p = 0.0458$).
For statistics, the Kruskal-Wallis test with multiple comparisons and Dunn's correction was applied.

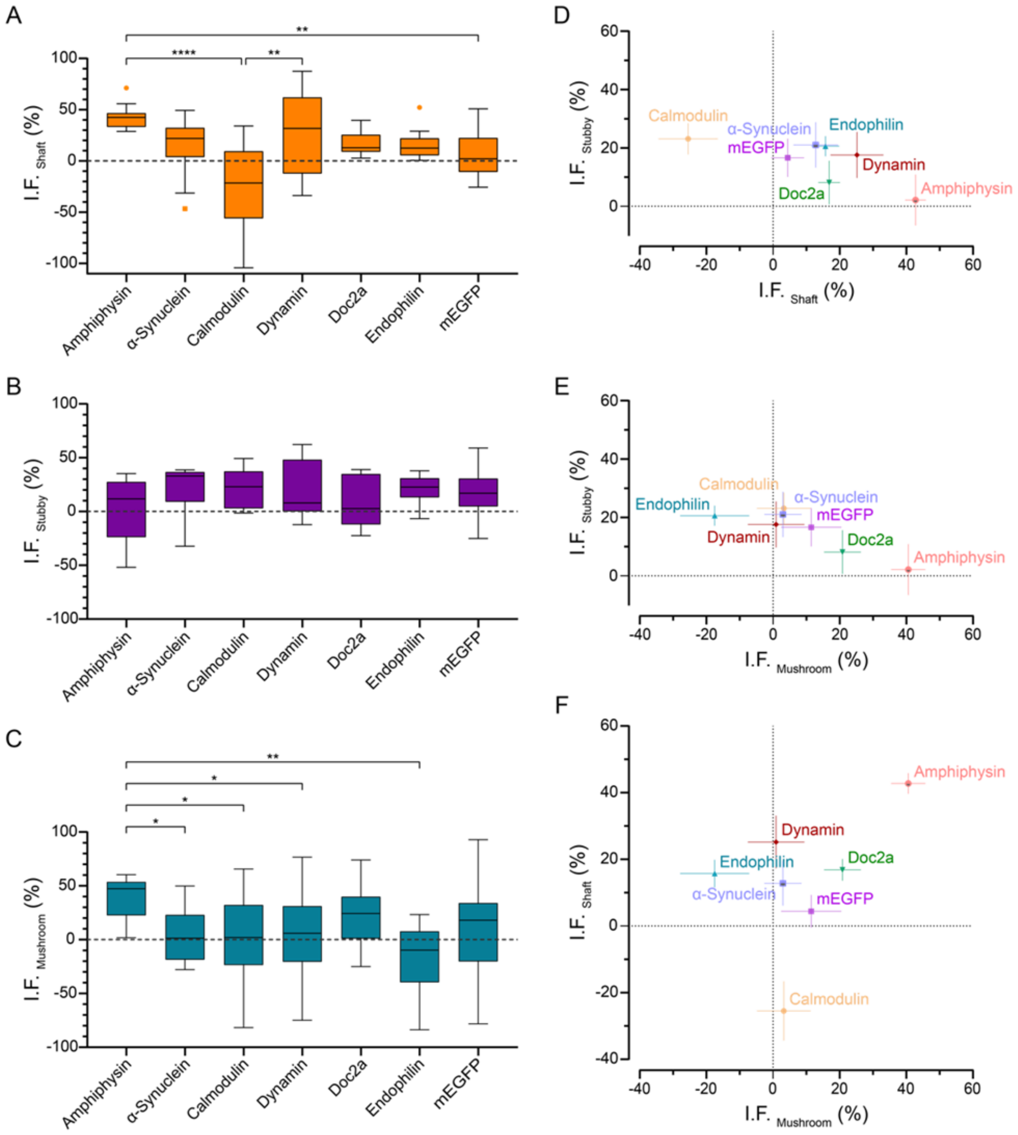

Figure S15: Immobile fractions of the different proteins in three postsynaptic compartments. Related to Figure 3.

(A-C) Box plots of immobile fractions (I.F.) in mushroom spines (A), stubby spines (B) and dendritic shafts (C) of α-synuclein, amphiphysin, calmodulin, doc2a, dynamin, endophilin and mEGFP. Asterisks indicate statistical significance after Kruskal-Wallis tests, followed by multiple comparisons with Dunn's correction. α = 0.05. **(D-F)** Immobile fractions (I.F.) of all analyzed proteins in mushroom spines and dendritic shafts (D), in mushroom spines and stubby spines (E), in stubby spines and shafts (F). Symbols show mean ± SEM.

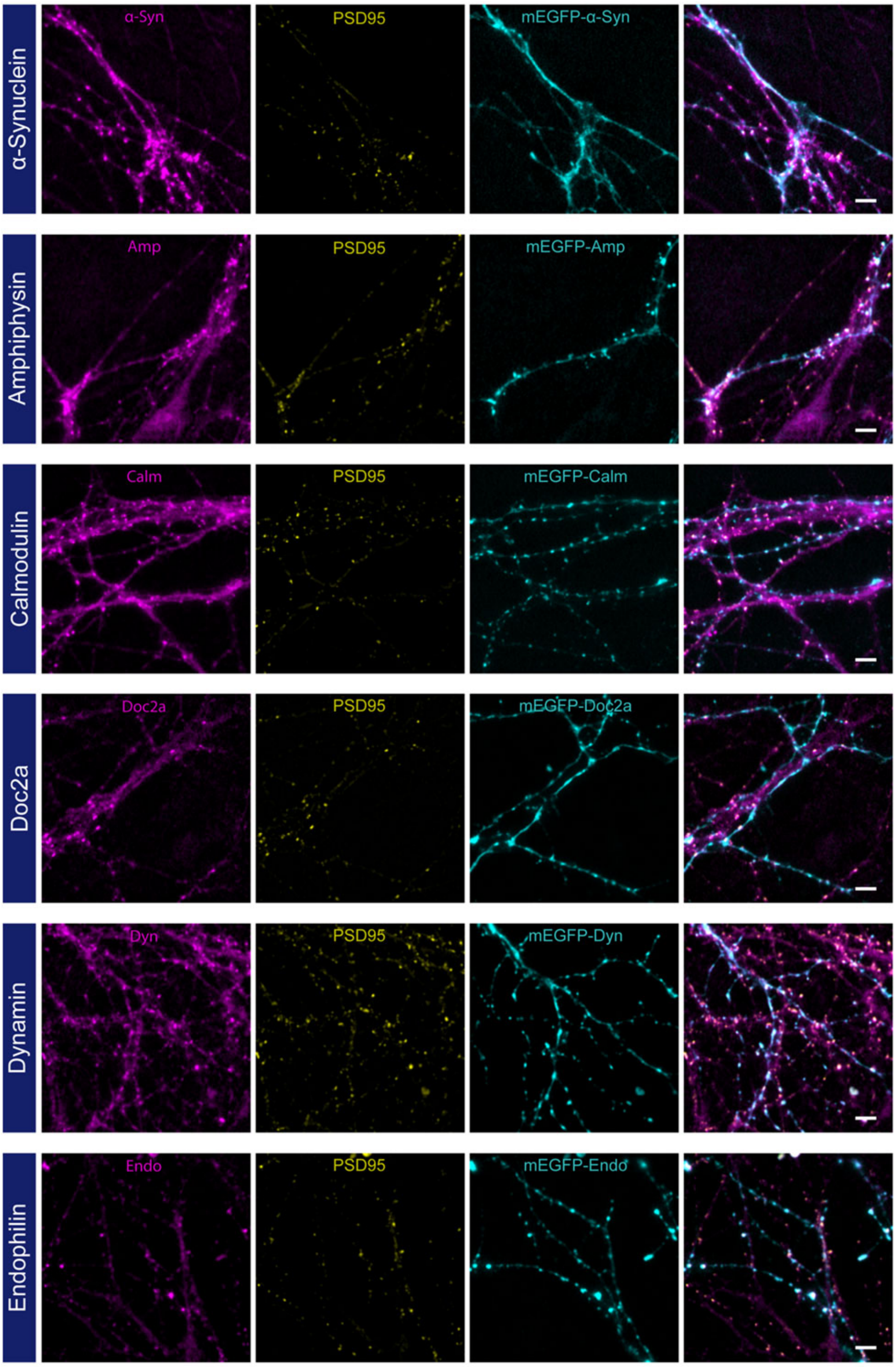

Figure S16: Representative images from neuronal cultures expressing mEGFP-tagged protein of interest. Related to STAR Methods. The protein of interest immunolabelling is shown in magenta, with PSD95 in yellow and the mEGFP signal in cyan. Scale bar: 5 µm.

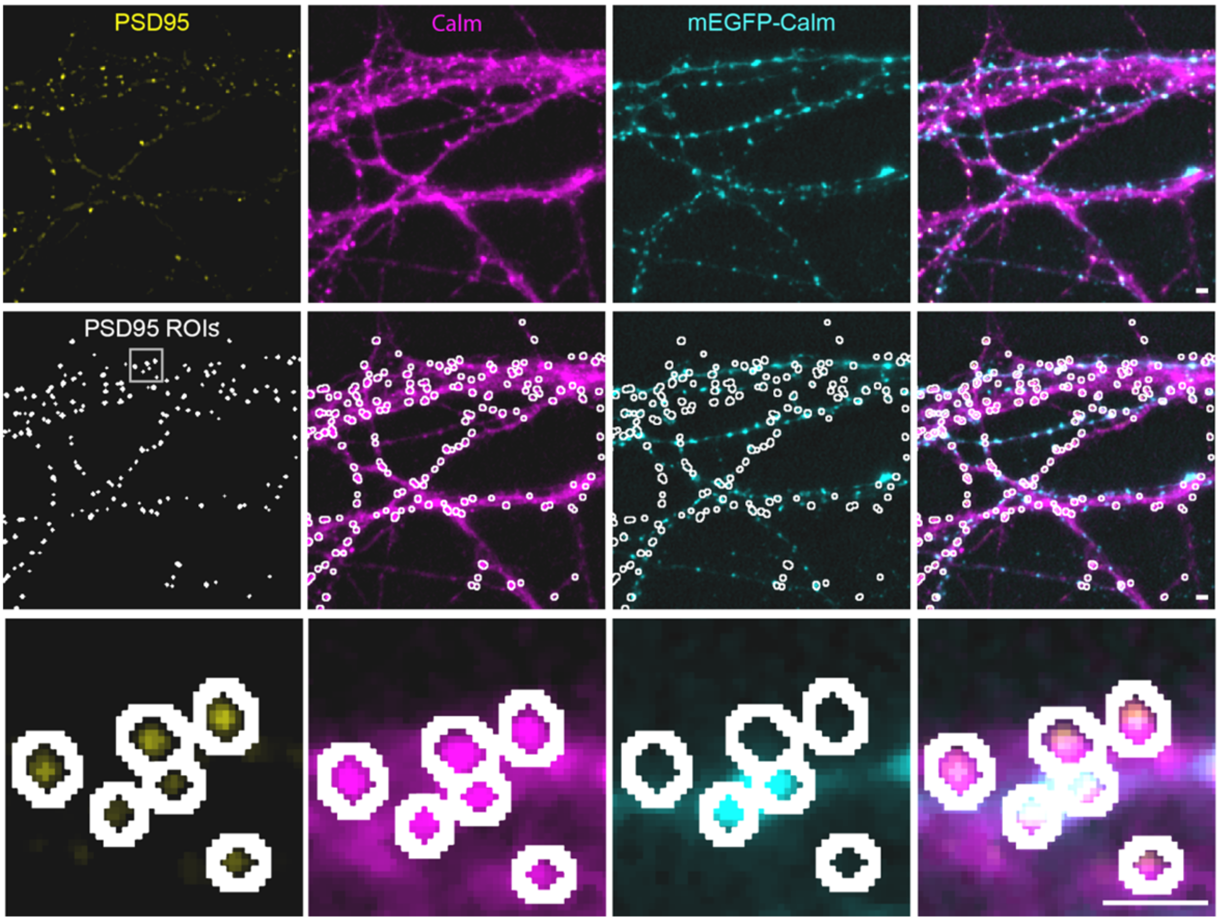

Figure S17: Exemplary images for explaining the overexpression analysis process. Related to STAR Methods.
Top: Original images used for the overexpression analysis, showing PSD95 (yellow), Calmodulin immunostaining**,** Calm, (magenta) and mEGFP-tagged Calmodulin, mEGFP-Calm (cyan). **Middle:** Relevant regions of interest (ROIs) for the overexpression analysis, in the form of PSD95-containing ROIs (white spots). These are generated by applying an empirically-derived threshold on the PSD95 image, in an automatic fashion. The intensity of the Calmodulin immunostaining (magenta) and mEGFP (cyan) is then measured in these ROIs, to measure the enhancement of the Calmodulin levels produced by the expression of mEGFP-Calmodulin. **Bottom:** Magnified delineated ROIs. For the overexpression quantification, Calmodulin immunostaining intensity was compared between mEGFP-positive and mEGFP-negative ROIs. Scale bar: 2 µm.

Table S1. Validations of the antibodies used in this study. Related to STAR Methods.

Antibody	Manufacturer	Identifier	Validation method	Reference
FluoTag®-X2 anti-PSD95	NanoTag Biotechnologies, Göttingen, Germany	N3702-Ab580-L	ICC, validated, as a marker of postsynaptic sites, by analyzing the signal localization and correlation to other PSD proteins	[S2]
Anti-alpha/beta Synuclein rabbit polyclonal antibody	Synaptic Systems, Göttingen, Germany	128 002	WB, ICC, IHC, EM	[S3,S4]
Anti-Amphiphysin rabbit polyclonal antibody	Synaptic Systems, Göttingen, Germany	120 002	WB, ICC, IHC, EM	[S4,S5]
Anti-Calmodulin rabbit monoclonal antibody	Novus Bioscience, Wiesbaden-Nordenstadt, Germany	NB110-55649	WB, ICC, IHC	[S4]
Anti-Doc 2a/b rabbit polyclonal antibody	Synaptic Systems, Göttingen, Germany	174 203	WB, ICC, IHC	[S4,S6]
Anti-Dynamin mouse monoclonal antibody	BD Bioscience, Heidelberg, Germany	610245	WB, ICC, IHC	[S4]
Anti-Endophilin 1 rabbit polyclonal antibody	Synaptic Systems, Göttingen, Germany	159 002	K.O. validated	[S7]
Anti-Synaptotagmin 1 mouse monoclonal antibody - luminal domain	Synaptic Systems, Göttingen, Germany	105 311	ICC, uptake, IP, WB	[S8–S10]

Table S2: Shapiro-Wilk normality test results for time constants (Taus) and immobile fractions (IFs) in different neuronal compartments for each protein of interest. Related to STAR Methods.

**Shapiro-Wilk test**		**Time constants (Taus)**			**Immobile Fractions (IFs)**		
**Protein of interest**	**Area**	**W**	**P value**	**Significance (α=0.05)**	**W**	**P value**	**Significance (α=0.05)**
**Amphiphysin**	Shaft	0,9277	0,2829	ns	0,8942	0,0931	ns
	Mushroom	0,9275	0,3155	ns	0,8565	0,0346	*
	Stubby	0,9144	0,2429	ns	0,8884	0,1123	ns
**α-Synuclein**	Shaft	0,9209	0,1744	ns	0,9078	0,1071	ns
	Mushroom	0,9604	0,6090	ns	0,9240	0,1520	ns
	Stubby	0,7759	0,0108	*	0,7740	0,0103	*
**Calmodulin**	Shaft	0,8987	0,0389	*	0,9557	0,4615	ns
	Mushroom	0,9261	0,1300	ns	0,9790	0,9201	ns
	Stubby	0,8997	0,1834	ns	0,9337	0,4491	ns
**Doc2a**	Shaft	0,9362	0,4771	ns	0,9177	0,2996	ns
	Mushroom	0,8196	0,0005	***	0,9575	0,3678	ns
	Stubby	0,9488	0,6539	ns	0,9011	0,2254	ns
**Dynamin**	Shaft	0,8880	0,0102	*	0,9238	0,0627	ns
	Mushroom	0,7682	0,0003	***	0,9607	0,5581	ns

	Stubby	0,9035	0,2036	ns	0,8883	0,1324	ns
**Endophilin**	Shaft	0,9165	0,2580	ns	0,8598	0,0486	*
	Mushroom	0,9068	0,2231	ns	0,8983	0,1760	ns
	Stubby	0,9448	0,4830	ns	0,9435	0,4645	ns
**mEGFP**	Shaft	0,9267	0,1504	ns	0,9581	0,5353	ns
	Mushroom	0,5421	<0,0001	****	0,9781	0,8723	ns
	Stubby	0,8934	0,1304	ns	0,9788	0,9783	ns

Supplementary References

1. Reshetniak, S., Ußling, J.-E., Perego, E., Rammner, B., Schikorski, T., Fornasiero, E.F., Truckenbrodt, S., Köster, S., and Rizzoli, S.O. (2020). A comparative analysis of the mobility of 45 proteins in the synaptic bouton. EMBO J *39*, e104596. 10.15252/embj.2020104596.

2. Shaib, A.H., Chouaib, A.A., Imani, V., Chowdhury, R., Georgiev, S.V., Mougios, N., Monga, M., Reshetniak, S., Mihaylov, D., Chen, H., et al. (2022). Expansion microscopy at one nanometer resolution. bioRxiv, 2022.08.03.502284. 10.1101/2022.08.03.502284.

3. Cole, N.B., DiEuliis, D., Leo, P., Mitchell, D.C., and Nussbaum, R.L. (2008). Mitochondrial translocation of α-synuclein is promoted by intracellular acidification. Exp Cell Res *314*, 2076–2089. 10.1016/j.yexcr.2008.03.012.

4. Wilhelm, B.G., Mandad, S., Truckenbrodt, S., Kröhnert, K., Schäfer, C., Rammner, B., Koo, S.J., Claßen, G.A., Krauss, M., Haucke, V., et al. (2014). Composition of isolated synaptic boutons reveals the amounts of vesicle trafficking proteins. Science *344*, 1023–1028. 10.1126/science.1252884.

5. Fuchs, M., Brandstätter, J., and Regus-Leidig, H. (2014). Evidence for a Clathrin-independent mode of endocytosis at a continuously active sensory synapse. Frontiers in Cellular Neuroscience *8*.

6. Schütz, D., Zilly, F., Lang, T., Jahn, R., and Bruns, D. (2005). A dual function for Munc-18 in exocytosis of PC12 cells. European Journal of Neuroscience *21*, 2419–2432. 10.1111/j.1460-9568.2005.04095.x.

7. Yang, Y., Chen, J., Guo, Z., Deng, S., Du, X., Zhu, S., Ye, C., Shi, Y.S., and Liu, J.-J. (2018). Endophilin A1 Promotes Actin Polymerization in Dendritic Spines Required for Synaptic Potentiation. Front Mol Neurosci *11*, 177. 10.3389/fnmol.2018.00177.

8. Chapman, E.R., and Jahn, R. (1994). Calcium-dependent interaction of the cytoplasmic region of synaptotagmin with membranes. Autonomous function of a single C2-homologous domain. Journal of Biological Chemistry *269*, 5735–5741. 10.1016/S0021-9258(17)37523-3.

9. Dankovich, T.M., Kaushik, R., Olsthoorn, L.H.M., Petersen, G.C., Giro, P.E., Kluever, V., Agüi-Gonzalez, P., Grewe, K., Bao, G., Beuermann, S., et al. (2021). Extracellular matrix remodeling through endocytosis and resurfacing of Tenascin-R. Nat Commun *12*, 7129. 10.1038/s41467-021-27462-7.

10. Truckenbrodt, S., Viplav, A., Jähne, S., Vogts, A., Denker, A., Wildhagen, H., Fornasiero, E.F., and Rizzoli, S.O. (2018). Newly produced synaptic vesicle proteins are preferentially used in synaptic transmission. The EMBO Journal *37*, e98044. 10.15252/embj.201798044.
